# Urinary chitinase 3-like protein 1 for early diagnosis of acute kidney injury: a prospective cohort study in adult critically ill patients

**DOI:** 10.1186/s13054-016-1192-x

**Published:** 2016-02-11

**Authors:** Jorien De Loor, Johan Decruyenaere, Kristel Demeyere, Lieve Nuytinck, Eric AJ Hoste, Evelyne Meyer

**Affiliations:** Department of Pharmacology, Toxicology and Biochemistry, Laboratory of Biochemistry, Ghent University, Faculty of Veterinary Medicine, Salisburylaan 133, B-9820 Merelbeke, Belgium; Department of Internal Medicine, Division of Intensive Care, Ghent University Hospital, Faculty of Medicine and Health Sciences , De Pintelaan 185, B-9000 Ghent, Belgium; Bimetra-Clinical Research Center Ghent, Ghent University Hospital, Faculty of Medicine and Health Sciences , De Pintelaan 185, B-9000 Ghent, Belgium; Research Foundation-Flanders, Egmontstraat 5, B-1000 Brussels, Belgium

**Keywords:** Acute kidney injury, Biological markers, Chitinase, Intensive care, Lipocalins

## Abstract

**Background:**

Acute kidney injury (AKI) occurs frequently and adversely affects patient and kidney outcomes, especially when its severity increases from stage 1 to stages 2 or 3. Early interventions may counteract such deterioration, but this requires early detection. Our aim was to evaluate whether the novel renal damage biomarker urinary chitinase 3-like protein 1 (UCHI3L1) can detect AKI stage ≥2 more early than serum creatinine and urine output, using the respective Kidney Disease | Improving Global Outcomes (KDIGO) criteria for definition and classification of AKI, and compare this to urinary neutrophil gelatinase-associated lipocalin (UNGAL).

**Methods:**

This was a translational single-center, prospective cohort study at the 22-bed surgical and 14-bed medical intensive care units (ICU) of Ghent University Hospital. We enrolled 181 severely ill adult patients who did not yet have AKI stage ≥2 based on the KDIGO criteria at time of enrollment. The concentration of creatinine (serum, urine) and CHI3L1 (serum, urine) was measured at least daily, and urine output hourly, in the period from enrollment till ICU discharge with a maximum of 7 ICU-days. The concentration of UNGAL was measured at enrollment. The primary endpoint was the development of AKI stage ≥2 within 12 h after enrollment.

**Results:**

After enrollment, 21 (12 %) patients developed AKI stage ≥2 within the next 7 days, with 6 (3 %) of them reaching this condition within the first 12 h. The enrollment concentration of UCHI3L1 predicted the occurrence of AKI stage ≥2 within the next 12 h with a good AUC-ROC of 0.792 (95 % CI: 0.726–0.849). This performance was similar to that of UNGAL (AUC-ROC of 0.748 (95 % CI: 0.678–0.810)). Also, the samples collected in the 24-h time frame preceding diagnosis of the 1^st^ episode of AKI stage ≥2 had a 2.0 times higher (95 % CI: 1.3–3.1) estimated marginal mean of UCHI3L1 than controls. We further found that increasing UCHI3L1 concentrations were associated with increasing AKI severity.

**Conclusions:**

In this pilot study we found that UCHI3L1 was a good biomarker for prediction of AKI stage ≥2 in adult ICU patients.

**Electronic supplementary material:**

The online version of this article (doi:10.1186/s13054-016-1192-x) contains supplementary material, which is available to authorized users.

## Background

Acute kidney injury (AKI) occurs in approximately half of adult critically ill patients [1–9]. Besides its recognized adverse effect on individual patient outcomes, both in the short- and long-term [1, 2, 4–10], AKI causes an important socioeconomic burden ensuing from its relationship with the development of chronic kidney disease (CKD) [11], and end-stage renal disease requiring renal replacement therapy (RRT) [12].

Current diagnostic and staging criteria for AKI were defined by the Kidney Disease | Improving Global Outcomes (KDIGO) AKI work group (Additional file 1: Table S1) and require monitoring of two surrogate glomerular filtration rate (GFR) markers, i.e., serum creatinine (SCr) and urine output (UO), and of the intervention RRT [13]. As renal stress and damage to the kidneys precede the observed decline in GFR [14], diagnostic AKI biomarker research in the last decade has focused on detection of these early signals [15–17]. Studies have shown that urinary biomarkers like neutrophil gelatinase-associated lipocalin (NGAL) [18–22], and recently the panel tissue inhibitor of metalloproteinases 2 (TIMP-2) and insulin-like growth factor-binding protein 7 (IGFBP7) [21, 23, 24], can detect AKI in critically ill patients earlier than SCr or UO, even when using the most sensitive KDIGO criteria. In addition, these biomarkers may also allow detection of other outcomes such as progression of AKI, use of RRT, development of CKD, and long-term mortality [25, 26]. The complexity of the AKI syndrome and the interest in detecting other outcomes highly warrants evaluation of yet further candidate renal stress or damage biomarkers aiming to find either complementary or - less realistically - truly superior ones.

Recently, our group discovered chitinase 3-like protein 3 (CHI3L3) as a novel candidate biomarker for sepsis-induced AKI, by urinary proteomics [27–29]. Validation with western blot analysis confirmed the presence of CHI3L3 in urine of septic mice with AKI, and its absence in urine of septic mice without AKI. In view of translational research [30], two other members of the number-18 glycoside hydrolase-family [31], i.e., chitinase 3-like protein 1 (CHI3L1) and acidic mammalian chitinase (CHIA), showed similar results. Subsequently, we found that CHI3L1 measured in urine was more discriminative for the presence of AKI in human septic patients than CHIA [28].

The number-18 glycoside hydrolase-family is special in that it comprises catalytically inactive proteins such as chitinase-like proteins (CLP, e.g., CHI3L1) in addition to catalytically active proteins such as chitinases [31]. These CLPs function as lectins because they can bind, but not hydrolyze, the glycan chitin, and therefore, represent the chi-lectin subfamily [32, 33].

The objective of this study was first to evaluate the diagnostic performance of the urinary biomarker CHI3L1 for early detection of AKI stage ≥2 in adult critically ill patients, and then to compare this performance to that of NGAL, which was chosen as the reference urinary biomarker.

## Methods

We followed recommendations for strengthening the reporting of observational studies in epidemiology (STROBE) (Additional file 1: Table S2) [34]. Details of the methods are provided in Additional file 1: Text S1 and Tables S3A-F. The methods for additional analyses not included in the manuscript are provided in Additional file 1: Text S2 and Tables S4A and B.

We will refer to AKI that was diagnosed and classified by KDIGO as AKI_SCr/UO_, while AKI_SCr_ will imply that the KDIGO UO criteria were discarded.

### Study population

We conducted a prospective cohort study at the 22-bed surgical and 14-bed medical intensive care units (ICU) of Ghent University Hospital from September 2012 till August 2014. The inclusion and exclusion criteria are shown in Table 1.

**Table 1:** Inclusion and exclusion criteria of the study

Inclusion criteria	Exclusion criteria
Age ≥18 y	AKI_SCr/UO_ stage ≥2 at time of enrollment^a^
Presence of both arterial and urinary catheter	
Expected ICU stay ≥48 h	
Respiratory SOFA score ≥2 (PaO_2_/FiO_2_ < 300) or cardiovascular SOFA score ≥1 (MAP <70 mmHg or on vasopressor(s) for at least 1 h)	CKD KDOQI stage 5 (GFR <15 ml/min/1.73 m^2^ or RRT)^b^
Written informed consent	

^a^Based on the Kidney Disease | Improving Global Outcomes (*KDIGO*) serum creatinine (*SCr*) or urine output (*UO*) criteria for acute kidney injury (*AKI*). ^b^Kidney Disease Outcomes Quality Initiative (*KDOQI*) [62]. *CKD* chronic kidney disease, *FiO*_*2*_ fraction of inspired oxygen, *GFR* glomerular filtration rate, *ICU* intensive care unit, *MAP* mean arterial pressure, *PaO*_*2*_ partial pressure of oxygen in arterial blood, *RRT* renal replacement therapy, *SOFA* Sepsis-related Organ Failure Assessment

### Ethics, consent and permissions

This study was approved by the Ethical Committee of the Ghent University Hospital (Belgian registration number of the study: B670201213147), and conducted in accordance with the declaration of Helsinki and in compliance with the Good Clinical Practice Guidelines. All patients or their legally authorized representatives provided written informed consent.

### Sample collection, sample handling, and data collection

Blood and urine were collected at enrollment. The large majority of patients i.e., 89 % was enrolled on either the first (28 %) or second (61 %) ICU day, while the minority i.e., 11 % was enrolled on either the third (9 %) or fourth (2 %) ICU day. Each subject was sampled a second time on the day of enrollment (d1) at 6 pm if the first collection was before noon. The subsequent sampling times were at 6 am and 6 pm on d2–4, and at 6 am on d5–7 (Additional file 1: Table S3A). This is similar to the methodology used in the hallmark study by Kashani et. al. [21]. If the patient was discharged from the ICU before d7, the study stopped.

These paired blood and urine samples were collected by standard methods and centrifuged by standard protocols. Serum and urine supernatants were stored at −80 °C and thawed at room temperature immediately prior to analysis. Clinical data needed to complete the individual clinical research files were extracted from the hospital records by study coordinators. Clinical data and samples were anonymized. JDL had access to the anonymized SCr and serum C-reactive protein data in order to determine the appropriate sample dilution for CHI3L1 measurement by enzyme-linked immunosorbent assay (ELISA) (Additional file 1: Table S3B). All other technicians were blinded to clinical data.

### Biomarker measurements

Creatinine and urinary NGAL (UNGAL) analyses were performed externally. Creatinine concentrations were measured with a kinetic rate-blanked Jaffé assay (commercial reagents, Roche Diagnostics, Basel, Switzerland) on a Cobas c502, while UNGAL concentrations were measured with a particle-enhanced turbidimetric immunoassay (ST001-3CA, BioPorto, Hellerup, Denmark) on a Modular P. The concentration of CHI3L1 was determined in-house with a sandwich ELISA (DC3L10, R&D Systems, Minneapolis, MN, USA).

Both urinary CHI3L1 (UCHI3L1) and UNGAL concentrations were statistically analyzed as such and after correction for urine dilution by using the ratio to urinary creatinine (UCr). The relative change in SCr measured at enrollment was defined as the ratio of the enrollment SCr to reference SCr. The UO after enrollment, defined as the mean UO in the first valid 6-h period after enrollment, was determined as the mean of the 6 UO values that were calculated each h in the first valid 6-h period after enrollment.

### Primary endpoint

The primary endpoint of the study was the development of AKI_SCr/UO_ stage ≥2 within 12 hours (h) after enrollment (Additional file 1: Table S1). Reference SCr was defined as the lowest SCr value within the last 3 months (mo) prior to enrollment. The details for calculation of UO are outlined in Additional file 1: Text S1.

### Secondary endpoints

Secondary endpoints of the study were: AKI_SCr/UO_ stage ≥2 within 24 h and 7 days after enrollment; AKI_SCr_ stage ≥2 within 12 h, 24 h and 7 days after enrollment (Additional file 1: Table S1).

### UCHI3L1 response to AKI

We compared samples that were collected in the 24 h preceding diagnosis of the first episode of AKI_SCr/UO_ stage ≥2 to those that were not followed by a first episode of AKI_SCr/UO_ stage ≥2 within the next 24 h. For this analysis, we excluded all samples collected in the period starting from diagnosis of the first episode of AKI_SCr/UO_ stage ≥2 till the end of the study.

For all 21 patients who developed AKI_SCr/UO_ stage ≥2 (reference time 0 h) within 7 days after enrollment, we documented the UCHI3L1 concentrations corresponding with the time points 24 h before, 12 h before, 12 h after, and 24 h after diagnosis of the first episode of AKI_SCr/UO_ stage ≥2. This allowed us to investigate the distribution of UCHI3L1 over time in patients with AKI_SCr/UO_ stage ≥2.

We also studied the distribution of UCHI3L1 in samples corresponding with different stages of severity of AKI_SCr/UO_. If the total study period of 7 days was completed, 11 serum and 11 urine samples were available per ICU patient. All available UCHI3L1 concentrations were classified according to their AKI_SCr/UO_ stage at that moment. As such, UCHI3L1 concentrations were divided into four groups: no AKI_SCr/UO_ at the time of sampling, and AKI_SCr/UO_ stages 1, 2, or 3 at the time of sampling.

### Statistical analysis

The primary analysis was based on comparison of the areas under the receiver-operating characteristics curves (AUC-ROC) of UCHI3L1 with those of UNGAL for predicting the defined endpoints, which was performed in MedCalc 15.2.1 (MedCalc Software, Oostende, Belgium). We also calculated Spearman’s coefficients of rank correlation with this program. In SPSS 22 (IBM, Armonk, NY, USA) we performed (1) mixed model analysis with log_10_(UCHI3L1) as the outcome variable; diagnosis of the first episode of AKI_SCr/UO_ stage ≥2 within 24 h after sampling, as the predictor variable; and patient as the random factor; (2) Fisher’s exact or the chi-square test - the 95 % confidence interval (CI) of a proportion was calculated with the Wilson procedure without correction for continuity [35, 36] - and the Mann–Whitney *U* test; (3) the Wilcoxon matched-pair signed-rank test; and (4) related-samples Friedman’s two-way analysis of variance by ranks. Box and whisker plots were generated in GraphPad Prism 5 (GraphPad Software, San Diego, CA, USA). For all analyses, two-sided *P* values <0.05 were considered statistically significant.

In Additional file 1: Text S1 and Tables S3C-F, we provide all details and also describe how the urinary biomarkers were introduced into the statistical programs.

## Results

The results of additional analyses not included in the manuscript are provided in Additional file 1: Text S2 and Tables S4C-I, Additional file 2: Figure S1, Additional file 3: Figure S2, Additional file 4: Figure S3, and Additional file 5: Figure S4.

### Patient characteristics and event rates

**Fig. 1:**
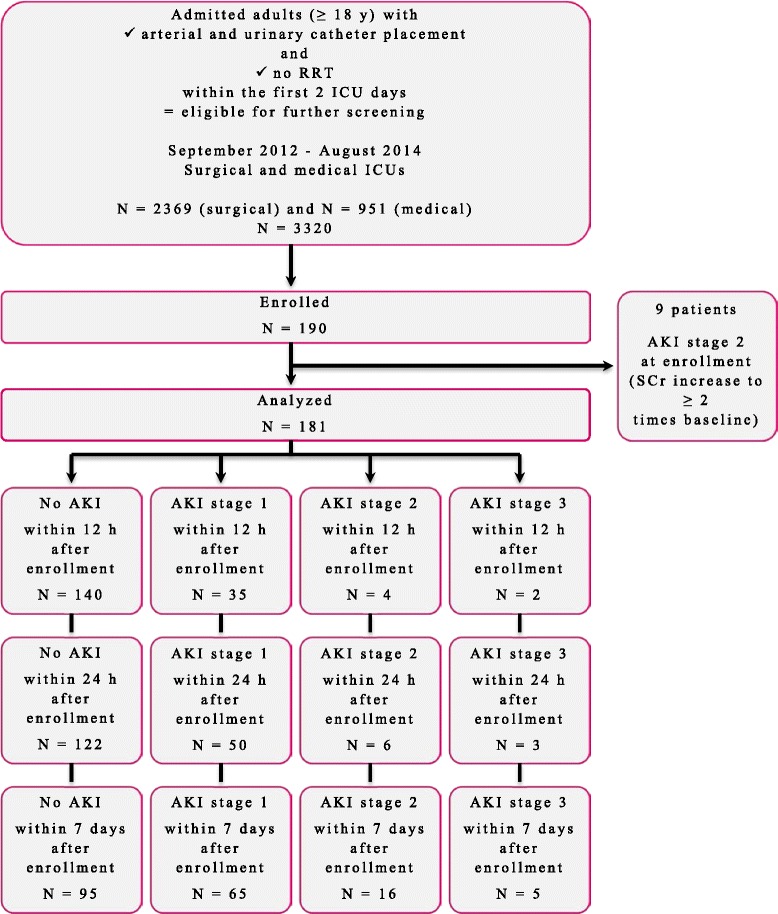
Flow diagram of the patient selection. Acute kidney injury (*AKI*) was defined and classified based on the Kidney Disease | Improving Global Outcomes serum creatinine (*SCr*) or urine output criteria. *ICU *intensive care unit, *RRT* renal replacement therapy, *y* year

The patient flow diagram is presented in Fig. 1. Of the 190 enrolled patients in our study cohort, 9 already fulfilled the SCr criteria for AKI stage 2 at enrollment and were therefore excluded. In this analysis cohort (n = 181), 21 patients (12 %) developed AKI_SCr/UO_ stage ≥2 within 7 days after enrollment. Within 24 h AKI_SCr/UO_ stage ≥2 was met by 9 patients (5 %) and within 12 h by 6 patients (3 %). In Table 2 the demographic information for these patients, either meeting or missing the primary endpoint, is depicted. Baseline characteristics were similar between both groups with the exception of an older age and a higher proportion of elective surgery in patients who developed AKI_SCr/UO_ stage ≥2 within 12 h after enrollment. In Table 3 the distribution of patients over different SCr and UO AKI stages that were maximally reached within 12 h, 24 h and 7 days after enrollment is shown.

**Table 2:** Demographic information for patients of the analysis cohort

	All patients	AKI_SCr/UO_ stage ≥ 2^a^	No AKI_SCr/UO_ stage ≥ 2^a^	*P* value
All patients	181 (100 %)	6 (3.3 %)	175 (96.7 %)	NA
		(1.5–7.0 %)	(93.0–98.5 %)	
Baseline characteristics				
Sex, male	114 (63.0 %)	4 (66.7 %)	110 (62.9 %)	1.000
	(55.7–69.7 %)	(30.0–90.3 %)	(55.5–69.7 %)	
Age^b^, y	60.0 (51.0–70.0)	70.5 (65.8–78.0)	59.0 (50.0–70.0)	0.040
Race, white	181 (100 %)	6 (100 %)	175 (100 %)	NA
	(97.9–100 %)	(61.0–100 %)	(97.9–100 %)	
Body mass index	24 (22–28)	25 (23–28)	24 (22–28)	0.594
Reference renal function				
SCr (mg/dl)	0.66 (0.52–0.79)	0.64 (0.55–0.69)	0.66 (0.51–0.79)	0.660
eGFR_MDRD_ (ml/min/1.73 m^2^)	119 (89–150)	120 (105–134)	119 (89–153)	0.975
eGFR_CKD-EPI_ (ml/min/1.73 m^2^)	102 (89–118)	98 (92–102)	103 (89–118)	0.392
Diabetes mellitus				0.787
Type I	2 (1.1 %)	0 (0.0 %)	2 (1.1 %)	
	(0.3–3.9 %)	(0.0–39.0 %)	(0.3–4.1 %)	
Type II	11 (6.1 %)	0 (0.0 %)	11 (6.3 %)	
	(3.4–10.6 %)	(0.0–39.0 %)	(3.5–10.9 %)	
Heart failure				1.000
NYHA class I	179 (98.9 %)	6 (100 %)	173 (98.9 %)	
	(96.1–99.7 %)	(61.0–100 %)	(95.9–99.7 %)	
NYHA class II	2 (1.1 %)	0 (0.0 %)	2 (1.1 %)	
	(0.3–3.9 %)	(0.0–39.0 %)	(0.3–4.1 %)	
NYHA class III	0 (0.0 %)	0 (0.0 %)	0 (0.0 %)	
	(0.0–2.1 %)	(0.0–39.0 %)	(0.0–2.1 %)	
NYHA class IV	0 (0.0 %)	0 (0.0 %)	0 (0.0 %)	
	(0.0–2.1 %)	(0.0–39.0 %)	(0.0–2.1 %)	
Myocardial infarction or cardiac arrest^c^	17 (9.4 %)	0 (0.0 %)	17 (9.7 %)	1.000
	(5.9–14.5 %)	(0.0–39.0 %)	(6.2–15.0 %)	
Malignancy^c^	17 (9.4 %)	1 (16.7 %)	16 (9.1 %)	0.451
	(5.9–14.5 %)	(3.0–56.4 %)	(5.7–14.3 %)	
ICU admission				
Referred from				0.063
Other hospital	42 (23.2 %)	0 (0.0 %)	42 (24.0 %)	
	(17.7–29.9 %)	(0.0–39.0 %)	(18.3–30.8 %)	
Emergency room	75 (41.4 %)	1 (16.7 %)	74 (42.3 %)	
	(34.5–48.7 %)	(3.0–56.4 %)	(35.2–49.7 %)	
Operating room	21 (11.6 %)	1 (16.7 %)	20 (11.4 %)	
	(7.7–17.1 %)	(3.0–56.4 %)	(35.8–50.3 %)	
Floor	43 (23.8 %)	4 (66.7 %)	39 (22.3 %)	
	(18.1–30.5 %)	(30.0–90.3 %)	(16.8–29.0 %)	
Reason				0.038
Medical	108 (59.7 %)	3 (50.0 %)	105 (60.0 %)	
	(52.4–66.5 %)	(18.8–81.2 %)	(52.6–67.0 %)	
Elective surgery	13 (7.2 %)	2 (33.3 %)	11 (6.3 %)	
	(4.2–11.9 %)	(9.7–70.0 %)	(3.5–10.9 %)	
Urgent surgery	60 (33.1 %)	1 (16.7 %)	59 (33.7 %)	
	(26.7–40.3 %)	(3.0–56.4 %)	(27.1–41.0 %)	
First day of study				
SOFA score (points)	9 (7–11)	11 (8–16)	9 (7–11)	0.189
Mechanical ventilation	122 (67.4 %)	5 (83.3 %)	117 (66.9 %)	0.665
	(60.3–73.8 %)	(43.6–97.0 %)	(59.6–73.4 %)	
Vasopressors	117 (64.6 %)	4 (66.7 %)	113 (64.6 %)	1.000
	(57.4–71.2 %)	(30.0–90.3 %)	(57.2–71.3 %)	
Infection	153 (84.5 %)	6 (100 %)	147 (84.0 %)	0.592
	(78.6–89.1 %)	(61.0–100 %)	(77.8–88.7 %)	
Infection ++^d^	122 (67.4 %)	6 (100 %)	116 (66.3 %)	0.179
	(60.3–73.8 %)	(61.0–100 %)	(59.0–72.9 %)	
Nephrotoxic drugs (before or at the first study day)				
ACE inhibitors	25 (13.8 %)	1 (16.7 %)	24 (13.7 %)	0.596
	(9.5–19.6 %)	(3.0–56.4 %)	(9.4–19.6 %)	
ARBs	5 (2.8 %)	0 (0.0 %)	5 (2.9 %)	1.000
	(1.2–6.3 %)	(0.0–39.0 %)	(1.2–6.5 %)	
Iodinated contrast media	81 (44.8 %)	2 (33.3 %)	79 (45.1 %)	0.693
	(37.7–52.0 %)	(9.7–70.0 %)	(38.0–52.5 %)	
Aminoglycosides	6 (3.3 %)	0 (0.0 %)	6 (3.4 %)	1.000
	(1.5–7.0 %)	(0.0–39.0 %)	(1.6–7.3 %)	
Vancomycine	15 (8.3 %)	0 (0.0 %)	15 (8.6 %)	1.000
	(5.1–13.2 %)	(0.0–39.0 %)	(5.3–13.7 %)	
Diuretics	45 (24.9 %)	3 (50.0 %)	42 (24.0 %)	0.164
	(19.1–31.6 %)	(18.8–81.2 %)	(18.3–30.8 %)	
Tacrolimus	2 (1.1 %)	1 (16.7 %)	1 (0.6 %)	0.065
	(0.3–3.9 %)	(3.0–56.4 %)	(0.1–3.2 %)	
Cyclosporine	1 (0.6 %)	0 (0.0 %)	1 (0.6 %)	1.000
	(0.1–3.1 %)	(0.0–39.0 %)	(0.1–3.2 %)	
NSAIDs (chronic)	10 (5.5 %)	1 (16.7 %)	9 (5.1 %)	0.292
	(3.0–9.9 %)	(3.0–56.4 %)	(2.7–9.5 %)	
Corticosteroids (chronic)	24 (13.3 %)	1 (16.7 %)	23 (13.1 %)	0.580
	(9.1–19.0 %)	(3.0–56.4 %)	(8.9–18.9 %)	

^a^Within 12 h after enrollment (primary endpoint); based on the Kidney Disease | Improving Global Outcomes (KDIGO) serum creatinine (SCr) or urine output (UO) criteria for acute kidney injury (AKI). ^b^Determined at the first day of the study. ^c^At time of hospital or ICU admission. ^d^Suspected bacterial infection, either leading to arterial hypotension or organ dysfunction, or leading to shock (Additional file 1: Table S4A). Data represent number (%) (95 % CI) and median (IQR) for categorical and continuous variables, respectively. *ACE* angiotensin converting enzyme, *ARB* angiotensin-II receptor blocker, *CI* confidence interval, *CKD-EPI* Chronic Kidney Disease Epidemiology Collaboration, *eGFR* estimated glomerular filtration rate, *ICU* intensive care unit, *IQR* interquartile range, *MDRD* Modification of Diet in Renal Disease, *NSAID* nonsteroidal anti-inflammatory drug, *NYHA* New York Heart Association, *SOFA* Sepsis-related Organ Failure Assessment

**Table 3:** Distribution of patients over different KDIGO serum creatinine and urine output acute kidney injury stages maximally reached within indicated observation periods

Time window	Maximum AKI stage Number (%)	UO 0^a^	UO 1^a^	UO 2^a^	UO 3^a^	Total in row
12 h	SCr 0^a^	140 (77)	░ 11 (6)	▒ 2 (1)	▓ 0 (0)	153 (85)
24 h		122 (67)	░ 19 (10)	▒ 4 (2)	▓ 0 (0)	145 (80)
7 days		95 (52)	░ 23 (13)	▒ 2 (1)	▓ 0 (0)	120 (66)
12 h	SCr 1^a^	░ 21 (12)	░ 3 (2)	▒ 0 (0)	▓ 0 (0)	24 (13)
24 h		░ 27 (15)	░ 4 (2)	▒ 0 (0)	▓ 0 (0)	31 (17)
7 days		░ 35 (19)	░ 7 (4)	▒ 3 (2)	▓ 0 (0)	45 (25)
12 h	SCr 2^a^	▒ 1 (1)	▒ 1 (1)	▒ 0 (0)	▓ 0 (0)	2 (1)
24 h		▒ 0 (0)	▒ 2 (1)	▒ 0 (0)	▓ 0 (0)	2 (1)
7 days		▒ 4 (2)	▒ 4 (2)	▒ 3 (2)	▓ 0 (0)	11 (6)
12 h	SCr 3^a^	▓ 1 (1)	▓ 1 (1)	▓ 0 (0)	▓ 0 (0)	2 (1)
24 h		▓ 2 (1)	▓ 1 (1)	▓ 0 (0)	▓ 0 (0)	3 (2)
7 days		▓ 4 (2)	▓ 1 (1)	▓ 0 (0)	▓ 0 (0)	5 (3)
12 h	Total in column	163 (90)	16 (9)	2 (1)	0 (0)	181 (100)
24 h		151 (83)	26 (14)	4 (2)	0 (0)	181 (100)
7 days		138 (76)	35 (19)	8 (4)	0 (0)	181 (100)

Patients with different stages of severity of acute kidney injury (*AKI*) diagnosed and classified by the Kidney Disease | Improving Global Outcomes (*KDIGO*) serum creatinine (*SCr*) or urine output (*UO*) criteria (*AKI*_*SCr/UO*_) are marked with a different shaded rectangle: the darker this shade, the worse the kidney injury. ^a^ *SCr 0* represents no AKI based on the KDIGO_SCr_ criteria. Likewise, *UO 0* represents no AKI based on the KDIGO_UO_ criteria. *SCr 1/2/3* represents AKI stage 1/2/3 based on the KDIGO_SCr_ criteria. Likewise, *UO 1/2/3* represents AKI stage 1/2/3 based on the KDIGO_UO_ criteria

### Biomarkers’ diagnostic performances

**Fig. 2:**
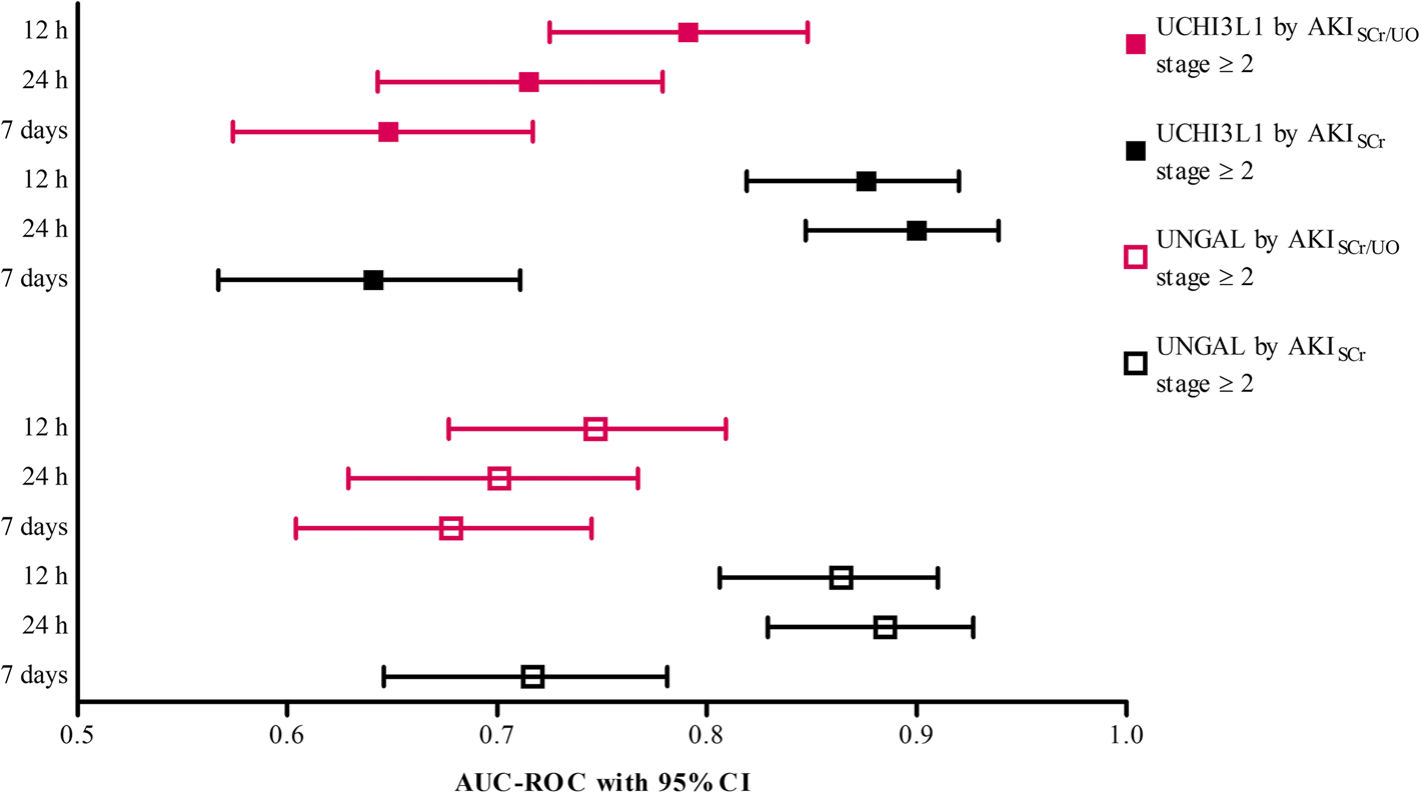
Area under the receiver-operating characteristics curve (*AUC-ROC*) with 95 % confidence interval (*CI*) for urinary chitinase 3-like protein 1 (*UCHI3L1*) and urinary neutrophil gelatinase-associated lipocalin (*UNGAL*) at enrollment for prediction of the six different endpoints. The *P* value for the difference in AUC-ROC between both biomarkers for predicting acute kidney injury (*AKI*) stage ≥2 based on the Kidney Disease | Improving Global Outcomes (*KDIGO*) serum creatinine (*SCr*) or urine output (*UO*) criteria (*AKI*_*SCr/UO*_) within 12 h after enrollment was 0.587. In the 24-h prediction window the *P* value was 0.823, and in the 7-d prediction window it was 0.522. Likewise, the *P* value for the difference in AUC-ROC between both biomarkers for predicting AKI_SCr_ stage ≥2 within 12 h after enrollment was 0.661. In the 24-h prediction window the *P* value was 0.495, and in the 7-d prediction window it was 0.099

The biomarkers UCHI3L1 and UNGAL, both measured at enrollment, were good predictors of the development of AKI_SCr/UO_ stage ≥2 within the next 12 h, with an AUC-ROC of 0.792 (95 % CI: 0.726–0.849) for UCHI3L1 and 0.748 (0.678–0.810) for UNGAL (*P* = 0.587; Fig. 2). When UO criteria were discarded, these AUC-ROCs improved to 0.877 (0.820–0.921) for UCHI3L1 and 0.865 (0.807–0.911) for UNGAL (*P* = 0.661). Extending our prediction window to 24 h slightly decreased the AUC-ROC for both biomarkers for predicting AKI_SCr/UO_ stage ≥2, while the AUC-ROC for predicting AKI_SCr_ stage ≥2 remained similar (Fig. 2). In the 7-d prediction window both biomarkers became poor predictors of AKI stage ≥2, irrespective of the definition used. To obtain 2 explicit clinical phenotypes, the AUC-ROC analyses were repeated excluding AKI stage 1 (comparing AKI stage ≥2 with no AKI). These showed essentially unchanged results for UCHI3L1 and UNGAL, both measured at enrollment (Additional file 1: Table S5).

Correction of the urinary biomarker concentrations for urine dilution by calculating the ratio to UCr, decreased the AUC-ROC for both biomarkers when diagnosing AKI stage ≥2 based on SCr or UO, while there was no difference observed when diagnosing AKI stage ≥2 based on SCr alone (Table 4).

**Table 4:** Comparison of areas under the receiver-operating characteristics curves

Biomarker measurement	Time window	AKI_SCr/UO_ stage ≥ 2^a^			AKI_SCr_ stage ≥ 2^b^		
		AUC-ROC	95 % CI	*P* value^c^	AUC-ROC	95 % CI	*P* value^c^
Number = 181							
▓ Enrollment UCHI3L1	12-h	▓ 0.792	▓ 0.726–0.849		▓ 0.877	▓ 0.820–0.921	
	24-h	▓ 0.716	▓ 0.644–0.780		▓ 0.901	▓ 0.848–0.940	
	Comparison with						
Enrollment UCr-corrected UCHI3L1	12-h	0.674	0.601–0.742	0.038	0.832	0.769–0.883	0.359
	24-h	0.587	0.511–0.659	0.005	0.861	0.802–0.908	0.308
Enrollment SCHI3L1	12-h	0.645	0.570–0.714	0.071	0.784	0.717–0.842	0.300
	24-h	0.548	0.472–0.622	0.022	0.830	0.767–0.881	0.324
Enrollment SCr	12-h	0.624	0.549–0.695	0.113	0.732	0.662–0.795	0.363
	24-h	0.639	0.565–0.709	0.406	0.766	0.697–0.826	0.278
Enrollment SCr to reference SCr ratio	12-h	0.690	0.618–0.757	0.274	0.806	0.741–0.861	0.614
	24-h	0.695	0.622–0.761	0.784	0.847	0.786–0.896	0.622
▓ Enrollment UNGAL	12-h	▓ 0.748	▓ 0.678–0.810		▓ 0.865	▓ 0.807–0.911	
	24-h	▓ 0.702	▓ 0.630–0.768		▓ 0.886	▓ 0.830–0.928	
	Comparison with						
Enrollment UCr-corrected UNGAL	12-h	0.622	0.547–0.693	0.007	0.795	0.729–0.851	0.123
	24-h	0.570	0.495–0.644	0.001	0.826	0.763–0.878	0.109
Number = 180^d^							
▓ Enrollment UCHI3L1	12-h	▓ 0.791	▓ 0.724–0.848		▓ 0.876	▓ 0.819–0.921	
	24-h	▓ 0.715	▓ 0.643–0.779		▓ 0.901	▓ 0.847–0.940	
	Comparison with						
UO after enrollment^e^	12-h	0.833	0.771–0.885	0.728	0.759	0.690–0.820	0.034
	24-h	0.808	0.743–0.863	0.438	0.795	0.728–0.851	0.022

^a^Based on the Kidney Disease | Improving Global Outcomes (*KDIGO*) serum creatinine (*SCr*) or urine output (*UO*) criteria for acute kidney injury (*AKI*). ^b^Based on the KDIGO SCr criteria for AKI. ^c^The *P* value is shown for the difference in the area under the receiver-operating characteristics curve (*AUC-ROC*). We always compared with the AUC-ROC of the urinary biomarker (UCHI3L1 or UNGAL; marked with a shaded rectangle). ^d^UO after enrollment could not be calculated in one patient. ^e^Defined as the mean UO in the first valid 6-h period after enrollment (in ml/kg/h). *CI* confidence interval, *SCHI3L1* serum chitinase 3-like protein 1, *UCHI3L1* urinary chitinase 3-like protein 1, *UCr* urinary creatinine, *UNGAL* urinary neutrophil gelatinase-associated lipocalin

In contrast to UCHI3L1, serum CHI3L1 (SCHI3L1) measured at enrollment was a poor predictor of AKI_SCr/UO_ stage ≥2 within the next 12 h, with an AUC-ROC of 0.645 (0.570–0.714). In the 24-h prediction window SCHI3L1 did not predict AKI_SCr/UO_ stage ≥2. Again, when diagnosing based on SCr alone, the AUC-ROC for SCHI3L1 improved, i.e., 0.784 (0.717–0.842) in the 12-h and 0.830 (0.767–0.881) in the 24-h prediction window (Table 4).

We additionally studied the discriminatory ability of the individual KDIGO parameters, i.e., (relative change in) SCr measured at enrollment and UO after enrollment (Table 4). The AUC-ROC was similar for UCHI3L1, SCr and UO. However, UCHI3L1 performed better than UO when diagnosing AKI stage ≥2 based on SCr alone (*P* = 0.034 in the 12-h and 0.022 in the 24-h prediction window). In addition, compared to SCr, there was a clear trend towards a better AUC-ROC for UCHI3L1, especially when diagnosing AKI stage ≥2 based on SCr or UO within 12 h after enrollment.

Combining both urinary biomarkers as the two-biomarker panel [UCHI3L1]•[UNGAL] did not improve the diagnostic performance of each of these single biomarkers for predicting either the 12-h or 24-h endpoints (Table 5). A positive relationship between these markers was observed, with a Spearman’s coefficient of rank correlation of 0.615 (0.515–0.698) (Additional file 1: Table S6).

**Table 5:** Comparison of AUC-ROC between the two-biomarker panel [urinary chitinase 3-like protein 1]•[urinary neutrophil gelatinase-associated lipocalin] and each of these single biomarkers

Two-biomarker panel	Time window	AKI_SCr/UO_ stage ≥2^a^			AKI_SCr_ stage ≥2^b^		
		AUC-ROC	95 % CI	*P* value	AUC-ROC	95 % CI	*P* value
Enrollment [UCHI3L1]•[UNGAL]	12 h	0.784	0.717–0.841	0.764^c^	0.874	0.817–0.919	0.879^c^
				0.522^d^			0.544^d^
	24 h	0.721	0.650–0.785	0.856^c^	0.899	0.845–0.939	0.877^c^
				0.622^d^			0.311^d^

^a^Based on the Kidney Disease | Improving Global Outcomes (*KDIGO*) serum creatinine (*SCr*) or urine output (*UO*) criteria for acute kidney injury (*AKI*). ^b^Based on the KDIGO SCr criteria for AKI. ^c^Difference in the area under the receiver-operating characteristics curve (*AUC-ROC*) between the two-biomarker panel and urinary chitinase 3-like protein 1 (*UCHI3L1*), both measured at enrollment. ^d^Difference in AUC-ROC between the two-biomarker panel and urinary neutrophil gelatinase-associated lipocalin (UNGAL), both measured at enrollment

With the Youden index a cutoff for UNGAL of 139 ng/ml was identified corresponding to the reference cutoff for this variable (>150 ng/ml [22]) (Additional file 1: Table S7).

### UCHI3L1 response to AKI

**Fig. 3:**
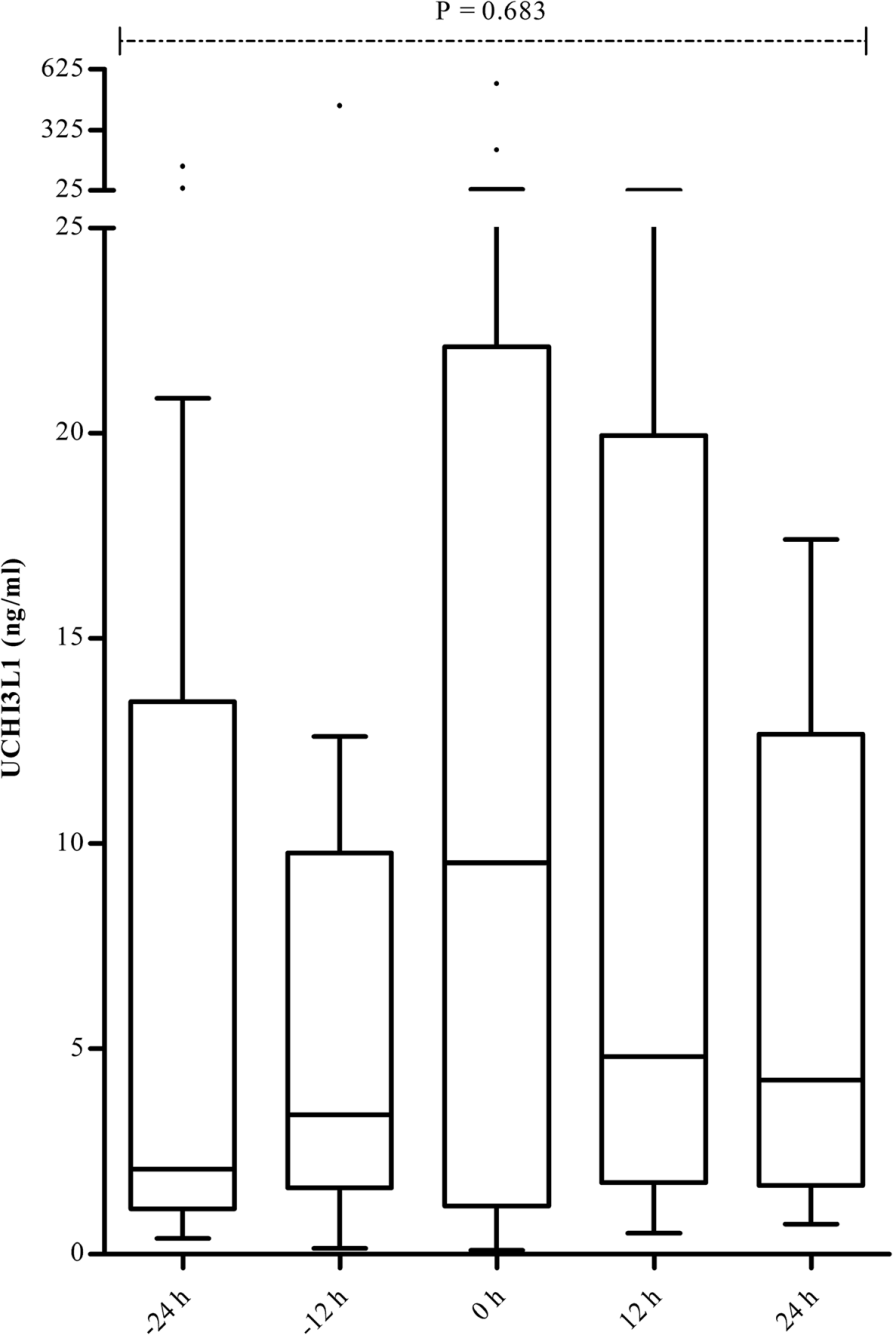
Distribution of urinary chitinase 3-like protein 1 (*UCHI3L1*) over time in patients who developed acute kidney injury (AKI) stage ≥2 based on the Kidney Disease | Improving Global Outcomes (KDIGO) serum creatinine (SCr) or urine output (UO) criteria (AKI_SCr/UO_) within 7 days after enrollment. The reference time 0 h represents time of diagnosis of the first episode of AKI_SCr/UO_ stage ≥2. At time −24 h, a UCHI3L1 concentration was available for 14 of these 21 patients. Likewise, 13 values were available at time −12 h, 19 at reference time 0 h, 14 at time 12 h, and 12 at time 24 h

Samples collected in the 24 h preceding diagnosis of the first episode of AKI_SCr/UO_ stage ≥2 had a 2.0 times higher (95 % CI: 1.3–3.1) estimated marginal mean of UCHI3L1 than those not followed by a first episode of AKI_SCr/UO_ stage ≥2 within the next 24 h. The general time–concentration profile of UCHI3L1 showed a trend for increasing concentrations from 24 h before, towards diagnosis of the first episode of AKI_SCr/UO_ stage ≥2 (reference time 0 h; Fig. 3). After reference time 0 h, median UCHI3L1 concentrations showed a decreasing trend.

**Fig. 4:**
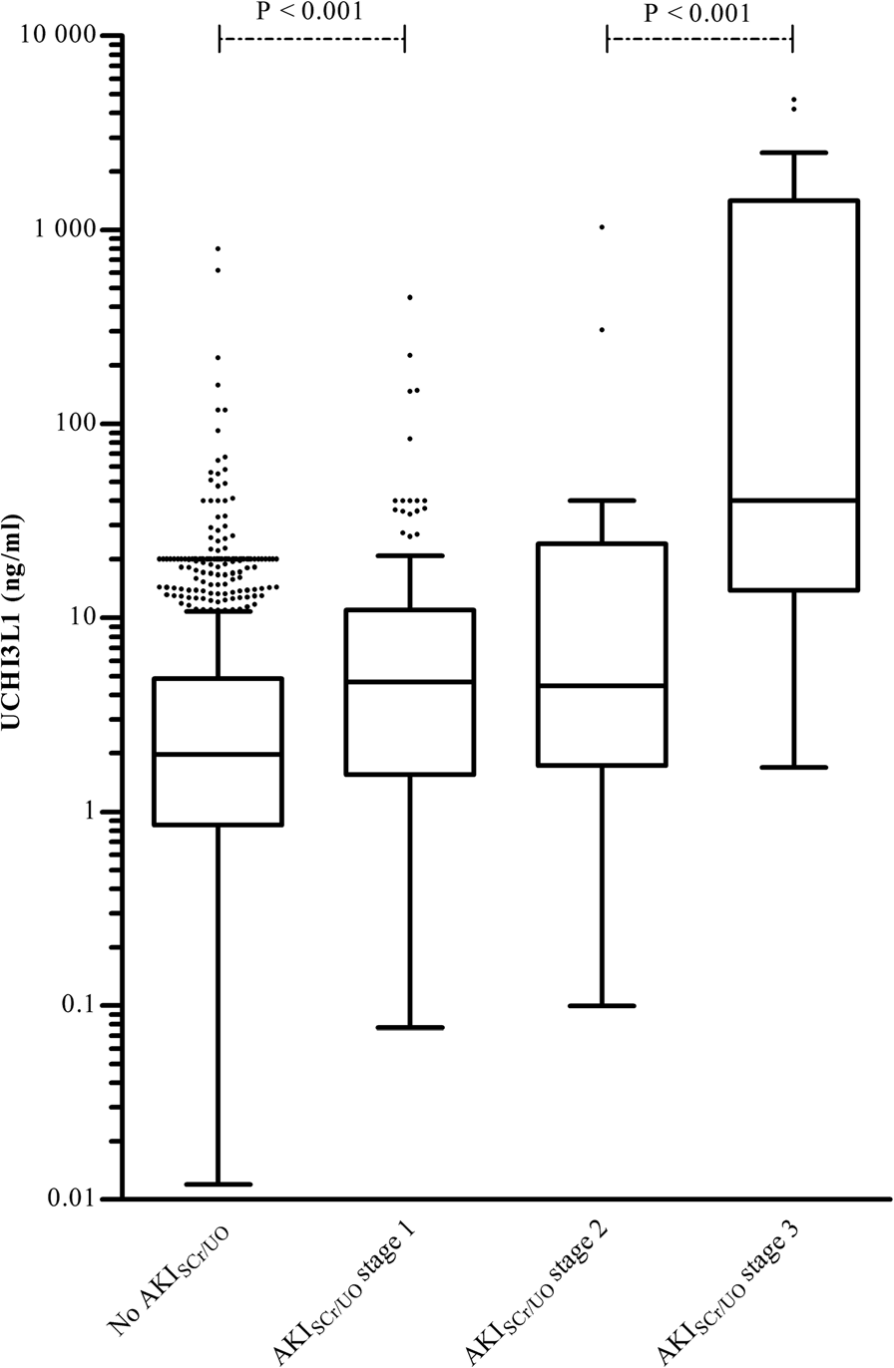
Distribution of urinary chitinase 3-like protein 1 (*UCHI3L1*) in samples corresponding with different stages of severity of acute kidney injury (*AKI*) based on the Kidney Disease | Improving Global Outcomes serum creatinine (*SCr*) or urine output (*UO*) criteria (*AKI*_*SCr/UO*_)

Samples corresponding with AKI_SCr/UO_ stage 1 at time of collection had higher UCHI3L1 concentrations than those corresponding with no AKI_SCr/UO_ at the time of collection (*P* <0.001) (Fig. 4). Stage 1 and stage 2 samples had similar UCHI3L1 concentrations (*P* = 0.514). Stage 3 samples again had higher UCHI3L1 concentrations than stage 2 samples (*P* <0.001).

## Discussion

We found that UCHI3L1 was a good biomarker for early detection of AKI stage ≥2 in adult critically ill patients admitted to an ICU, with a performance similar to that of UNGAL.

These findings may have important clinical and socioeconomic impact. Increasing severity of AKI is associated with increased risk of worse patient and kidney outcomes [1, 2, 4–10]. Importantly, observational and also intervention studies showed that early AKI management can counteract AKI deterioration, and is associated with lower mortality and less RRT dependence at discharge [37–44]. Consequently, even earlier identification of AKI using a biomarker may have a much stronger effect on these outcomes.

Both UCHI3L1 and UNGAL better predicted AKI stage ≥2 that was defined based on SCr alone versus based on SCr or UO. These two urinary proteins are biomarkers of renal stress or damage [45, 46], while SCr and UO are GFR surrogates. However, UO is much more sensitive to decline in GFR, and therefore is probably associated with less renal stress or damage than SCr, which is supported by studies reporting that UO-based AKI classes are associated with a lower ICU/hospital mortality than SCr-based ones [9, 47, 48]. This may explain the better AUC-ROCs when considering SCr alone for diagnosis. The findings by Macedo et al. [49], who reported similar ICU mortality for exclusively UO^+^ AKI patients (8.8 %) and (non)oliguric SCr^+^ AKI patients (10.4 %), appear contradictory to previous findings [9, 47, 48]. However, severity of AKI was greater in exclusively UO^+^ patients: >60 % of these patients were stage 2, while >70 % of the (non)oliguric SCr^+^ patients were stage 1 [49]. We also observed a partial overlap in UCHI3L1 between AKI_SCr/UO_ stage 1 and stage 2 samples, indicating heterogeneity of AKI severity within KDIGO classes, which can be partly explained by the different impact of meeting the defined criteria for either UO alone, or SCr alone, or both SCr and UO [9]. This could also clarify the decreased performance of UCHI3L1 and UNGAL at enrollment for prediction of AKI_SCr/UO_ stage ≥2 within the next 24 h. The majority of the extra AKI_SCr/UO_ stage ≥2 patients in the 24 h observation period fulfilled UO criteria only. These patients, therefore, probably had less renal stress or damage, and consequently a low biomarker signal. Another explanation could be that the hit leading to AKI is following the biomarker measurement. This may more likely occur when the observation period is longer [21].

The observation that the AUC-ROC for the individual KDIGO parameters, i.e., SCr and UO, were similar to those of UCHI3L1 for detection of AKI stage ≥2, warrants discussion. First, when comparing the AUC-ROC for UO and UCHI3L1, we must take into account that although the measurement of UO started at enrollment, it was only completed 6 h later than the time at which UCHI3L1 was measured. Second, renal stress or damage may not always be reflected by decline in GFR; vice versa, a decline in GFR may not always reflect renal stress or damage. This may lead to underestimation of the diagnostic performance of UCHI3L1 in our study.

We found a trend for increased UCHI3L1 concentrations in the 24 h preceding AKI, and for decreased concentrations afterwards. However, it should be emphasized that after meeting AKI_SCr/UO_ stage ≥2, the individual time–concentration profiles of AKI_SCr/UO_ differed widely between patients: some remained in the same severity stage, some deteriorated and others ameliorated (data not shown). The number of patients observed in this pilot study also precludes firm conclusions.

This is the first translational study demonstrating that UCHI3L1 predicts the occurrence of AKI stage ≥2 in adult critically ill patients [28]. Schmidt et al. independently showed that UCHI3L1 predicts the occurrence of delayed graft function in adult patients who receive deceased-donor kidney transplants [46]. In their preclinical study, these authors reported that the transcription of the *CHI3L1* gene is significantly upregulated in the mouse kidney after ischemia/reperfusion (I/R) injury with increased excretion of its protein in urine. These mRNA and protein levels correlated with the degree of kidney injury and were at earliest measured on the first day after I/R, when SCr values had already peaked. Recently, the same group also studied a cohort of hospitalized patients who had AKI, and found that UCHI3L1 was associated with the composite outcome of AKI progression and in-hospital death [50].

We must speculate on the source of CHI3L1. Upon renal stress or damage, this protein is secreted by macrophages within the kidney [46], while NGAL is secreted by specific cells of the distal nephron [45]. Another source for the urine component of NGAL is the circulating plasma pool [51]. We speculate that the same is true for CHI3L1 as this protein has an apparent molecular weight of ±39–40 kDa [52, 53], and as within the group of patients with no AKI (in the 7-d prediction window) a concomitant very high level of SCHI3L1 was observed more in those with an increased than with a normal UCHI3L1 at enrollment (Additional file 1: Table S8). Additionally, we speculate that CHI3L1 binds to the megalin receptor for tubular reabsorption. This implies that NGAL and CHI3L1 can each indirectly affect the urinary concentration of the other, as they are then competitors [51].

Similar to NGAL, CHI3L1 is also stored in the secondary granules of circulating neutrophils [54–56]. This could implicate that in the urine of patients with a urinary tract infection (UTI), CHI3L1 is increased too [57]. Although data on UCHI3L1 in UTI patients are missing, proteome profiling of human neutrophils suggests that this issue is less relevant for UCHI3L1 [56], which agrees with the reported 12 pg NGAL and 0.16 pg CHI3L1 per leukocyte [57, 58].

Surprisingly, only in 2013 He et al. investigated the possibility that the CLP CHI3L1 mediates its biological effects through receptor binding, and identified interleukin-13 receptor α2 as the binding partner [59]. These biological effects include inhibition of apoptosis in renal epithelial cells [46, 59], and inhibition of pyroptosis and interleukin-1β production in macrophages [59, 60]. These innate immune cells play an important role in both kidney injury and repair [61].

Our study has important limitations. First, this is a single-center study conducted in surgical and medical ICUs. Although the baseline characteristics of patients, the observed outcomes, and the NGAL cutoff based on the Youden index suggest that the patients included are representative of ICUs in developed Western countries, these data remain to be confirmed in other centers and in different types of ICU. Second, only a limited number of patients reached the primary endpoint, which can be partly explained by selection bias, i.e., not asking (legally authorized representatives of) the most critically ill patients for consent. Yet, this is a typical and hence, rather unavoidable feature of prospective studies like this [21]. The restricted period for observation of AKI stage ≥2 certainly contributes to the low event rate as well. Therefore, we included all 21 patients (12 %) who developed AKI_SCr/UO_ stage ≥2 within 7 days after enrollment in the mixed model analysis. Third, following the KDIGO guidelines [13], reference SCr was defined as the lowest SCr value within the last 3 mo prior to enrollment. This method is prone to bias, as blood draws for SCr measurement tend to be performed more often when patients are in hospital or sick, thereby not reflecting true baseline kidney function. Fourth, we did not measure urinary [TIMP-2]•[IGFBP7], a two-biomarker panel found to be superior to UNGAL [21, 23, 24], because it was not available at the start of our study.

## Conclusions

In summary, we demonstrated that UCHI3L1 measured in critically ill patients admitted to an ICU, predicted the occurrence of AKI stage ≥2 within a 12-h or 24-h observation period. The results of this pilot study need confirmation in different settings and in larger patient cohorts.

## Key messages

- The urinary proteome contains very relevant information: following an acute renal attack, CHI3L1 will appear in urine, indicating damage to the kidneys.
- In this pilot cohort of critically ill patients admitted to an ICU, the UCHI3L1 response predicted development of AKI stage ≥2 within a 12-h or 24-h period.
- The higher the concentration of UCHI3L1, the greater the severity of AKI.

## Additional files

Additional file 1:
**Supplemental data.** This file provides all details on the study methods (Supplemental Text S1 and Tables S3A-F) and on additional analyses not included in the manuscript (Supplemental Text S2, Tables S4A-I and Figure Legends S1-S4). It also contains the Supplemental Tables S1, S2, S5-S8, to which we refer in the manuscript. (PDF 628 kb) Additional file 2: Figure S1. Area under the receiver-operating characteristics curve (*AUC-ROC*) with 95 % confidence interval (CI) of urinary chitinase 3-like protein 1 (*UCHI3L1*) (**a**) and urinary neutrophil gelatinase-associated lipocalin (*UNGAL*) (**b**) at enrollment for predicting acute kidney injury (*AKI*) stage ≥2 based on the Kidney Disease | Improving Global Outcomes (KDIGO) serum creatinine (*SCr*) or urine output (*UO*) criteria (*AKI*_*SCr/UO*_) within 12 h in different subgroups of patients. (TIF 434 kb) Additional file 3: Figure S2. Area under the receiver-operating characteristics curve (*AUC-ROC*) with 95 % confidence interval (CI) of urinary chitinase 3-like protein 1 (*UCHI3L1*) (**a**) and urinary neutrophil gelatinase-associated lipocalin (*UNGAL*) (**b**) at enrollment for predicting acute kidney injury (*AKI*) stage ≥2 based on the Kidney Disease | Improving Global Outcomes (KDIGO) serum creatinine (*SCr*) criteria (*AKI*_*SCr*_) within 24 h in different subgroups of patients. (TIF 436 kb) Additional file 4: Figure S3. Distribution of urinary chitinase 3-like protein 1 (*UCHI3L1*) (**a**) and urinary neutrophil gelatinase-associated lipocalin (UNGAL) (**b**) at enrollment in the eight selected subgroups of patients who did not develop acute kidney injury (AKI) based on the Kidney Disease | Improving Global Outcomes serum creatinine (*SCr*) or urine output (*UO*) criteria (*No AKI*_*SCr/UO*_) within 7 days after enrollment, compared to the distribution in all patients with no AKI_SCr/UO_ within 12 h after enrollment, and in all those maximally reaching AKI_SCr/UO_ stages 1, 2, or 3 within 12 h after enrollment. (TIF 715 kb) Additional file 5: Figure S4. Distribution of urinary chitinase 3-like protein 1 (*UCHI3L1*) (**a**) and urinary neutrophil gelatinase-associated lipocalin (*UNGAL*) (**b**) at enrollment in the eight selected subgroups of patients who did not develop acute kidney injury (*AKI*) based on the Kidney Disease | Improving Global Outcomes serum creatinine (*SCr*) criteria (*No AKI*_*SCr*_) within 7 days after enrollment, compared to the distribution in all patients with no AKI_SCr_ within 24 h after enrollment, and in all those maximally reaching AKI_SCr_ stages 1, 2, or 3 within 24 h after enrollment. (TIF 716 kb)

## Abbreviations

AKI: acute kidney injury
AKI_SCr_: AKI that was diagnosed and classified by the Kidney Disease | Improving Global Outcomes serum creatinine criteria
AKI_SCr/UO_: AKI that was diagnosed and classified by the Kidney Disease | Improving Global Outcomes serum creatinine or urine output criteria
AUC-ROC: area under the receiver-operating characteristics curve
CHI3L1: chitinase 3-like protein 1
CHI3L3: chitinase 3-like protein 3
CHIA: acidic mammalian chitinase
CI: confidence interval
CKD: chronic kidney disease
CLP: chitinase-like protein
d1: day of enrollment
ELISA: enzyme-linked immunosorbent assay
GFR: glomerular filtration rate
h: hour
I/R: ischemia/reperfusion
ICU: intensive care unit
IGFBP7: insulin-like growth factor-binding protein 7
KDIGO: Kidney Disease | Improving Global Outcomes
mo: month
NGAL: neutrophil gelatinase-associated lipocalin
RRT: renal replacement therapy
SCHI3L1: serum chitinase 3-like protein 1
SCr: serum creatinine
STROBE: strengthening the reporting of observational studies in epidemiology
TIMP-2: tissue inhibitor of metalloproteinases 2
UCHI3L1: urinary chitinase 3-like protein 1
UCr: urinary creatinine
UNGAL: urinary neutrophil gelatinase-associated lipocalin
UO: urine output
UTI: urinary tract infection
