# Supplementary material for: Urinary chitinase 3-like protein 1 for early diagnosis of acute kidney injury: a prospective cohort study in adult critically ill patients
Source: Crit Care. 2016 Feb 11;20:38. doi: 10.1186/s13054-016-1192-x (PMC4750195; doi:10.1186/s13054-016-1192-x)
Supplement: Additional file 1: — Supplemental data. This file provides all details on the study methods (Supplemental Text S1 and Tables S3A-F) and on additional analyses not included in the manuscript (Supplemental Text S2, Tables S4A-I and Figure Legends S1-S4). It also contains the Supplemental Tables S1, S2, S5-S8, to which we refer in the manuscript. (PDF 628 kb) [file 13054_2016_1192_MOESM1_ESM.pdf]

ADDITIONAL FILE 1 SUPPLEMENTAL DATA.

INDEX

SUPPLEMENTAL TEXTS 4
TEXT S1: COMPLETE METHODS 4
Sample collection and handling 4
Blood (serum) 4
Urine 4
Biomarker measurements 5
Creatinine 5
CHI3L1 5
NGAL 6
UO calculation 7
Statistical analysis 7
Review and adjustment of CHI3L1 concentrations before input in statistical programs 8
Step 1: Evaluation of the standard curve of the ELISA 8
Step 2: Re-analysis of samples with a concentration (not adjusted for dilution) outside the validated dynamic range of the curve 8
Step 3: Adjustment of CHI3L1 concentrations of optimally diluted samples with OD_{sample} <$OD_{62.5}$ 8
Adjustment of UNGAL concentrations before input in statistical programs 9
TEXT S2: ADDITIONAL ANALYSES 10
METHODS 10
Subgroup analyses 10
Additional AUC-ROC analyses 10
Validation of the analytical stability of CHI3L1 11
Short-term stability of CHI3L1 in serum and urine before centrifugation 11
Combined long-term and freeze-thaw stability of CHI3L1 in urine 11
Partial in-house validation of the CHI3L1 ELISA 11
Within-run precision or intra-assay variability 11
Between-run precision or inter-assay variability 12
Calculation of the LOD and LOQ 12
Linearity check for urine 12
RESULTS 14
Biomarkers' diagnostic performances in subgroups 14
Additional diagnostic performances 14
Stability of CHI3L1 in serum and urine 15
Partial in-house validation of the CHI3L1 ELISA 15

CONCLUSIONS REGARDING THE VALIDATION OF THE ANALYTICAL STABILITY OF CHI3L1 AND THE PARTIAL IN-HOUSE VALIDATION OF THE CHI3L1 ELISA 17

LIST OF ABBREVIATIONS 18

SUPPLEMENTAL REFERENCES 19

SUPPLEMENTAL TABLES 21

Table S1 Kidney Disease: Improving Global Outcomes definition and classification of acute kidney injury.[a] 21

Table S2 STrengthening the Reporting of OBservational studies in Epidemiology statement - checklist of items that should be included in the reports of cohort studies.[a] 22

Table S3A Two representative work schemes for sample centrifugation in weekends. 25

Table S3B Dilution of serum and urine samples for the initial measurement of chitinase 3-like protein 1 by enzyme-linked immunosorbent assay. 26

Table S3C Evaluation of the assured dynamic range of the chitinase 3-like protein 1 enzyme-linked immunosorbent assay standard curve. 27

Table S3D Re-analysis of samples with a concentration (not adjusted for dilution) outside the validated dynamic range of the chitinase 3-like protein 1 enzyme-linked immunosorbent assay standard curve. 28

Table S3E Adjustment of chitinase 3-like protein 1 concentrations of optimally diluted samples with optical $density_{sample}$ < optical $density_{62.5}$. 29

Table S3F Adjustment of urinary neutrophil gelatinase-associated lipocalin concentrations before input in statistical programs. 31

Table S4A Definition of suspected bacterial infection, of arterial hypotension, of organ dysfunction, and of shock. 32

Table S4B Experimental setup for evaluation of between-run precision or inter-assay variability by the manufacturer. 33

Table S4C Areas under the receiver-operating characteristics curves with 95% confidence interval of urinary chitinase 3-like protein 1 and urinary neutrophil gelatinase-associated lipocalin at enrollment for prediction of the additional endpoints. 34

Table S4D Short-term stability of chitinase 3-like protein 1 in serum and urine before centrifugation. 35

Table S4E Combined long-term and freeze-thaw stability of chitinase 3-like protein 1 in urine. 36

Table S4F Within-run precision or intra-assay variability of the chitinase 3-like protein 1 enzyme-linked immunosorbent assay for standard points. 37

Table S4G Within-run precision or intra-assay variability of the chitinase 3-like protein 1 enzyme-linked immunosorbent assay for serum samples. 39

Table S4H Within-run precision or intra-assay variability of the chitinase 3-like protein 1 enzyme-linked immunosorbent assay for urine samples. 40

Table S4I Assessment of the enzyme-linked immunosorbent assay linearity for urinary chitinase 3-like protein 1. 41

Table S5: Sharpened clinical phenotype analysis with areas under the receiver-operating characteristics curves of urinary chitinase 3-like protein 1 and urinary neutrophil gelatinase-associated lipocalin at enrollment. 42

Table S6: Spearman’s coefficients of rank correlation for UCHI3L1 and UNGAL, both measured at enrollment, in the total analysis cohort, in subgroups separated by AKI stage at enrollment, and in subgroups separated by AKI stage within 12-h and 24-h after enrollment. 43

Table S7: Youden index with the associated criterion value of the urinary biomarker. 44

Table S8: Proportion of patients with a concomitant very high serum chitinase 3-like protein 1 in the groups of patients who did not develop acute kidney injury and either presented with or without an increased urinary chitinase 3-like protein 1 at enrollment. 45

SUPPLEMENTAL FIGURE LEGENDS 46

Figure S1 Area under the receiver-operating characteristics curve (AUC-ROC) with 95% confidence interval (CI) of (A) urinary chitinase 3-like protein 1 (UCHI3L1) and (B) urinary neutrophil gelatinase-associated lipocalin (UNGAL) at enrollment for predicting acute kidney injury (AKI) stage ≥2 based on the Kidney Disease: Improving Global Outcomes (KDIGO) serum creatinine (SCr) or urine output (UO) criteria ($AKI_{SCr/UO}$) within 12-h in different subgroups of patients. 46

Figure S2 Area under the receiver-operating characteristics curve (AUC-ROC) with 95% confidence interval (CI) of (A) urinary chitinase 3-like protein 1 (UCHI3L1) and (B) urinary neutrophil gelatinase-associated lipocalin (UNGAL) at enrollment for predicting acute kidney injury (AKI) stage ≥2 based on the Kidney Disease: Improving Global Outcomes (KDIGO) serum creatinine (SCr) criteria (AKI_{SCr}) within 24-h in different subgroups of patients. 46

Figure S3 Distribution of (A) urinary chitinase 3-like protein 1 (UCHI3L1) and (B) urinary neutrophil gelatinase-associated lipocalin (UNGAL) at enrollment in the 8 selected subgroups of patients who did not develop acute kidney injury (AKI) based on the Kidney Disease: Improving Global Outcomes serum creatinine (SCr) or urine output (UO) criteria (no-$AKI_{SCr/UO}$) within 7-d after enrollment, compared to the distribution in all 12-h no-$AKI_{SCr/UO}$ patients, and in all those maximally reaching $AKI_{SCr/UO}$ stages 1, 2, or 3 within 12-h after enrollment. 47

Figure S4 Distribution of (A) urinary chitinase 3-like protein 1 (UCHI3L1) and (B) urinary neutrophil gelatinase-associated lipocalin (UNGAL) at enrollment in the 8 selected subgroups of patients who did not develop acute kidney injury (AKI) based on the Kidney Disease: Improving Global Outcomes serum creatinine (SCr) criteria (no-AKI_{SCr}) within 7-d after enrollment, compared to the distribution in all 24-h no-AKI_{SCr} patients, and in all those maximally reaching AKI_{SCr} stages 1, 2, or 3 within 24-h after enrollment. 47

SUPPLEMENTAL TEXTS

TEXT S1: COMPLETE METHODS

Sample collection and handling

Blood (serum)

Approximately 6 ml of blood was obtained via an indwelling arterial line at each study-specific sampling moment. Blood was collected in clot activator collection tubes (Venosafe 6 ml, ref. VF-106SAS, Terumo Europe, Leuven, BE) for serum. After clotting at 4°C (in weekends: storage up to ±40-hours (h; Table S3A) at 4°C based on our stability results (Table S4D)), serum samples were centrifuged (Heraeus Labofuge 400 R, swinging bucket rotor with round bucket, Thermo Fisher Scientific, Waltham, MA) at 4°C and 1992 x g for 15-minutes (min). The supernatant was divided into 4 aliquots: 1 for chitinase 3-like protein 1 (CHI3L1), 1 for creatinine (Cr), and 2 as backup. These eppendorf tubes containing the supernatant were immediately stored at -80°C. No preservatives were added. Samples were thawed at room temperature immediately prior to analysis and vortexed before pipetting.

Urine

Approximately 5-10 ml of urine was obtained via an indwelling bladder catheter at each study-specific sampling moment. Urine was collected directly from the catheter (never from the collection bag) via the needle-free port-system in a standard (non-coated) transport container that can also be used as centrifuge tube for sediment recovery (Urine Monovette 10 ml, ref. 10.252, Sarstedt, Nümbrecht, DE). Urine was immediately (in weekends: storage up to ±40-h (Table S3A) at 4°C based on our stability results (Table S4D)) centrifuged (Heraeus Labofuge 400 R, swinging bucket rotor with round bucket, Thermo Fisher Scientific, Waltham, MA) at 4°C and 1029 x g for 10-min. The supernatant was divided into 5 aliquots: 1 for CHI3L1, 1 for neutrophil gelatinase-associated lipocalin (NGAL), 1 for Cr, and 2 as

backup. These eppendorf tubes containing the supernatant were immediately stored at -80°C. No preservatives were added. Samples were thawed at room temperature immediately prior to analysis and vortexed before pipetting. Eppendorf tubes that still contained visible sediment were very shortly (<15-seconds) centrifuged (Heraeus Biofuge Fresco, Thermo Fisher Scientific, Waltham, MA) at 4°C and 7697 x g before pipetting.

Biomarker measurements

Creatinine

Creatinine concentrations were measured in the 24-h laboratory of Ghent University Hospital with a kinetic rate-blanked Jaffé assay (commercial reagents, Roche Diagnostics, Basel, CH) on a Cobas c502.

All samples were analyzed within 6-months (mo) after collection (median: 3-mo; interquartile range (IQR): 2-4 mo) complying with the reported stability: when stored without preservatives at -22 respectively -25°C, Cr was stable for 15-years (y) in urine [1], and for 25-y in serum [2].

CHI3L1

The concentration of CHI3L1 was determined in-house by *J. De Loor*, *K. Demeyere*, and *K. Van Nuffel* with a sandwich enzyme-linked immunosorbent assay (ELISA; Human Chitinase 3-like 1 Quantikine ELISA Kit, ref. DC3L10, R&D Systems, Minneapolis, MN).The standard procedure that was followed when measuring CHI3L1 by ELISA is as follows. All samples and reagents were brought to room temperature. Samples and standards requiring dilution were accordingly prepared using calibrator diluents (1/200 or 1/500 dilution for serum; 1/5 or 1/10 dilution for urine; Table S3B). To each well pre-coated with a rat monoclonal antibody against recombinant human CHI3L1 we added 100 µl of assay diluents followed by 50 µl of the appropriate sample or standard. This mixture was allowed to react for 2-h at room temperature. Each well was then aspirated and washed 4 times before adding 200 µl of a

horseradish peroxidase-conjugated goat polyclonal antibody against recombinant human CHI3L1. After another incubation time of 2-h at room temperature, each well was again aspirated and washed 4 times. We then added 200 µl of substrate solution per well, which consisted of 100 µl of hydrogen peroxide and 100 µl of tetramethylbenzidine. This mixture was incubated in the dark for 30-min. Finally, 50 µl of stop solution was added per well after which the optical density (OD) of each well was measured with a microplate reader (Multiskan MS microplate reader, Thermo Fisher Scientific, Waltham, MA) set at 450 nm. The correction wavelength was set at 550 nm. The serum and urinary CHI3L1 concentrations were calculated with a microplate analysis program (DeltaSoft JV, Biomettalics, Princeton Junction, NJ). The 4-parameter logistic (4PL) model was chosen for curve fitting, as described by the manufacturer.

All samples were analyzed within 13-mo after collection (median: 7-mo; IQR: 3-10 mo). When stored without preservatives (personal communication with Johansen, JS) at -80°C, serum CHI3L1 (SCHI3L1) is stable for 8-y [3]. Additionally, we showed that urinary CHI3L1 (UCHI3L1) may even be measured after a second thawing step within at least 30-mo after the first freezing (Table S4E).

NGAL

The concentration of urinary NGAL (UNGAL) was measured in the clinical chemistry laboratory of Ghent University Hospital with a particle-enhanced turbidimetric immunoassay (PETIA; NGAL Test, ref. ST001-3CA, BioPorto, Hellerup, DK) on a Modular P. The standard procedure that was followed when measuring UNGAL by PETIA is as follows. All samples and reagents were brought to room temperature. After calibrating and running the controls 150 µl of sample was provided in a specific sample cup, as described by the manufacturer. To read over the measuring principle of this PETIA, we refer to other literature [4].

All NGAL analyses were performed in batch in November 2014. Storage at -80°C for 2-y without preservatives has been shown not to affect UNGAL [5].

UO calculation

For urine output (UO) calculations we accepted a margin of 10% under the 1-h block. Therefore, all urine volume measurements over a period less than 0.9-h (54-min) were first counted up with the following measurement. Then, we redistributed all blocks ≥1.8- and <2.7-h into 2 blocks, and all blocks ≥2.7- and ≤3-h into 3 blocks, generating blocks of ≥0.9- and <1.8-h. Blocks >3-h were considered as unreliable for redistribution and UO calculation.

Statistical analysis

The primary analysis was based on comparison of the areas under the receiver-operating characteristics curves (AUC-ROC) of UCHI3L1 with those of UNGAL for predicting the defined endpoints, which was performed in MedCalc 15.2.1 (MedCalc Software, Oostende, BE). The method by DeLong et al. was selected for calculation of the standard error of both the AUC-ROC and the difference between 2 AUC-ROCs [6]. For the AUC-ROC, a binomial exact 95% confidence interval (CI) was calculated. This program also lists the Youden index [7], defined as the maximum of [sensitivity plus specificity minus 1], with its associated criterion for each AUC-ROC. We also calculated Spearman's coefficients of rank correlation with this program.

In SPSS 22 (IBM, Armonk, NY) we performed:

(a) Mixed model analysis with $\log_{10}$(UCHI3L1) as outcome variable, diagnosis of the 1st episode of acute kidney injury (AKI) stage ≥2 based on the Kidney Disease: Improving Global Outcomes (KDIGO) serum Cr (SCr) or UO criteria ($AKI_{SCr/UO}$) within 24-h after sampling as predictor variable, and patient as random factor.

(b) Unpaired comparison of a variable between 2 independent samples. Categorical variables were analyzed with Fisher's exact or the chi-square test, and continuous variables with the

nonparametric Mann-Whitney U test. The SPSS ‘Descriptives’ menu uses Method 6 from the article by Hyndman and Fan for calculation of the IQR [8]. Additionally, we calculated the 95% CI for a proportion using the Wilson procedure without a correction for continuity [9, 10].

(c) Paired comparison of a continuous variable between 2 related samples (Tables S4D and E) using the Wilcoxon matched-pair signed-rank test.

(d) Paired comparison of a continuous variable between >2 related samples using the related-samples Friedman’s two-way analysis of variance by ranks.

Box and whisker plots were generated in GraphPad Prism 5 (GraphPad Software, San Diego, CA), which also uses Method 6 for calculation of the 1st and 3rd quartile [8]. The method by Tukey was selected for drawing of the whiskers [11].

For all analyses, 2-sided P values <0.05 were considered significant.

We will now describe how the urinary biomarkers were introduced into the statistical models.

Review and adjustment of CHI3L1 concentrations before input in statistical programs

Remark: CHI3L1 concentrations are expressed in **pg/ml** in these raw data.

Step 1: Evaluation of the standard curve of the ELISA

Only if the coefficient of determination (R^2) ≥0.995, there is a good fit of the 7 standard points in the 4PL model. Additionally, we evaluated whether the assured dynamic range of the standard curve was in fact as dynamic as guaranteed (Table S3C).

Step 2: Re-analysis of samples with a concentration (not adjusted for dilution) outside the validated dynamic range of the curve

The details of this standard procedure are outlined in Table S3D.

Step 3: Adjustment of CHI3L1 concentrations of optimally diluted samples with OD_{sample} $<OD_{62.5}$

Table S3E in detail outlines the standard procedure that was followed in this case. Note that, based on the acceptable recovery limits (70-130%), the back-calculated Concentration(Conc)$_{62.5}$ ranges from 43.8-81.3 pg/ml.

Adjustment of UNGAL concentrations before input in statistical programs

In the ‘performance data and application note for Roche Modular P’ *(http://ngal.com/media/30866/the_ngal_test_roche_modular_p_ivd.pdf)* can be read that the limit of quantification (LOQ) of the NGAL Test was determined to be 25 ng/ml on this analyzer model. As the limit of detection (LOD) was not tested on this model we were advised (personal communication with BioPorto) to use the LOQ that was estimated on the Roche Hitachi 917 *(http://ngal.com/media/30857/the_ngal_test_roche_hitachi_917_ivd.pdf)*, which was 12 ng/ml. The measured UNGAL concentrations were adjusted based on these reported limits (Table S3F).

TEXT S2: ADDITIONAL ANALYSES

METHODS

Subgroup analyses

We evaluated the biomarkers' diagnostic performances for predicting the primary endpoint as well as the 24-h AKI_{SCr} secondary endpoint in subgroups of patients. The selected variables used for grouping were: age, baseline estimated GFR (eGFR) calculated with the Chronic Kidney Disease Epidemiology Collaboration (CKD-EPI) formula, reason for ICU admission, patient's location prior to ICU admission, Sepsis-related Organ Failure Assessment (SOFA) score at d1, and presence of suspected bacterial infection, either leading to arterial hypotension or organ dysfunction, or leading to shock (Table S4A), at d1.

Additionally, we used the same grouping variables in both patients who did not develop $AKI_{SCr/UO}$ within 7-d after enrollment and those who did not develop AKI_{SCr} within 7-d after enrollment. The distribution of UCHI3L1 and UNGAL at enrollment in selected subgroups of 7-d no-$AKI_{SCr/UO}$ patients was plotted against the distribution in all 12-h no-$AKI_{SCr/UO}$ patients and in all those maximally reaching $AKI_{SCr/UO}$ stages 1, 2, or 3 within 12-h after enrollment. Likewise, the distribution of these biomarkers at enrollment in selected subgroups of 7-d no-AKI_{SCr} patients was plotted against the distribution in all 24-h no-AKI_{SCr} patients and in all those maximally reaching AKI_{SCr} stages 1, 2, or 3 within 24-h after enrollment.

Additional AUC-ROC analyses

Additional endpoints of the study were: $AKI_{SCr/UO}$ stage ≥1 within 12-h and 24-h after enrollment; AKI_{SCr} stage ≥1 within 12-h and 24-h after enrollment. As these endpoints include AKI stage 1, we additionally excluded the patients with AKI stage 1 at enrollment from the analysis cohort (n=181). The number of patients in the resulting sub-cohorts was 158 ($AKI_{SCr/UO}$) and 160 (AKI_{SCr}).

Validation of the analytical stability of CHI3L1

Short-term stability of CHI3L1 in serum and urine before centrifugation

Høgdall et al. found no change in the CHI3L1 concentration when serum samples were left on the clot at 4°C for 24-h before centrifugation, however, after 72-h SCHI3L1 was significantly increased [12]. We further tested the stability of CHI3L1 in serum (n=2) and also in urine (n=4) of intensive care unit (ICU) patients when stored at 4°C for 6-h (urine), 24-h, and 48-h before centrifugation by comparing these concentrations with those of the immediately centrifuged samples and calculating the mean coefficient of variation (CV).

Combined long-term and freeze-thaw stability of CHI3L1 in urine

As mentioned above, SCHI3L1 is stable for 8-y when stored at -80°C without preservatives. Høgdall et al. additionally showed that repetitive freezing and thawing of serum samples up to 8 times does not influence the concentration of SCHI3L1 [12].

For our study the most relevant stability feature concerning UCHI3L1 was whether UCHI3L1 measured in an aliquot that was thawed for the first time stayed stable after refreezing followed by thawing for the second time. Indeed, this was the protocol when UCHI3L1 fell outside the range of the standard curve at the first measurement. To evaluate this combined stability we compared those concentrations measured after the second thawing with those measured after the first thawing and calculated the mean CV. Total freezing times (=time between first freezing and second thawing), specifically for this stability evaluation, ranged from 6-30 mo (n=2 for 6-mo, 12-mo, 18-mo, 24-mo, and 30-mo).

Partial in-house validation of the CHI3L1 ELISA

Within-run precision or intra-assay variability

Of the 101 ELISA runs performed for CHI3L1 measurement (with ELISA kits from 6 different lots), 31 runs had replicate analyses of at least 1 sample or standard. These replicate samples were divided into 3 (serum) or 2 (urine) groups, i.e. low, intermediate (serum), and

high, covering the analytical range of the standard curve (0.06 - 4.00 ng/ml). Note that for urine there was no intermediate group as there were no replicates available of urine samples with a UCHI3L1 concentration >1.0 and ≤2.5 ng/ml. Replicate analyses of the 0.25, 0.50 and 1.00 ng/ml standards were consistently performed as these constitute the middle 3 points of the standard curve. The intra-assay between-lot CV was calculated as the weighted mean of the mean intra-assay within-lot CVs.

Between-run precision or inter-assay variability

Upon inquiry it appeared that the inter-assay CVs reported by the manufacturer were generated using 40 different assays that were divided between 4 technicians. Each of them performed one ELISA per day. The 40 assays consisted of 2 different matched sets of reagents, just like 2 different kit lots (Table S4B), so lot-to-lot variation was taken into account. Therefore, this part of the validation process was not repeated in-house. Three samples (type not specified) with a known CHI3L1 concentration (±0.50, ±1.00, and ±2.00 ng/ml) were analyzed. The reported mean inter-assay between-lot CV was 5.3% for the ±0.50 ng/ml sample, 5.8% for the ±1.00 ng/ml sample, and 6.9% for the ±2.00 ng/ml sample. The mean of these 3 CVs is 6.0%.

Calculation of the LOD and LOQ

The minimal detectable dose or LOD was determined by adding 2.6 [13] standard deviations (SD) to the mean OD value of 10 replicates of the zero standard (i.e. calibrator diluents) and calculating the corresponding concentration (DeltaSoft JV, Biomettalics, Princeton Junction, NJ). Likewise, the minimal quantifiable dose or LOQ was determined by adding 10 [14] SDs to the mean OD value of 10 replicates of the zero standard and calculating the corresponding concentration.

Linearity check for urine

The linearity of the assay was assessed by the manufacturer using samples from apparently healthy volunteers. Because our study population consisted of critically ill patients we rechecked the linearity for the specimen type urine. More specifically, we wanted to investigate 'how far undiluted' we could go as urinary components linked with severe illness may possibly interfere with the CHI3L1 measurement by ELISA. Therefore, undiluted urine was not tested and designated as unsuitable. The reference for our serial dilution experiment (1/2 - 1/4 - 1/8 - 1/16) was the 1/2 diluted sample. The relationship between the measured (not adjusted for dilution) and the expected (1/2 as reference) analyte concentration was investigated by linear regression analysis (GraphPad Prism 5, GraphPad Software, San Diego, CA).

RESULTS

Biomarkers’ diagnostic performances in subgroups

Diagnostic performance at enrollment for prediction of $AKI_{SCr/UO}$ stage ≥2 within the next 12-h, could be calculated in 9 of the 12 subgroups (Additional File 2: Supplemental Figure S1). Likewise, diagnostic performance at enrollment for prediction of AKI_{SCr} stage ≥2 within the next 24-h, could be calculated in 8 of the 12 subgroups (Additional File 3: Supplemental Figure S2). As for UCHI3L1, this biomarker showed decreased diagnostic performance - defined as an AUC-ROC <the lowest border of the 95% CI in the analysis cohort - for predicting the primary endpoint in patients either with a medical reason for ICU admission, or referred from either an emergency room, or operating room, or other hospital at ICU admission, or with a SOFA score <12 at d1 [15]. Its performance for predicting the 24-h AKI_{SCr} secondary endpoint was decreased in patients either ≥65-y old, or with a SOFA score ≥12 at d1. As for UNGAL, this biomarker showed decreased diagnostic performance for predicting the primary endpoint in patients either ≥65-y old, or with a medical reason for ICU admission, or referred from the floor at ICU admission. In the latter 2 subgroups UNGAL could not predict the primary endpoint. Its performance for predicting the 24-h AKI_{SCr} secondary endpoint was decreased in patients ≥65-y old.

We found that patients referred from the floor at ICU admission who did not develop $AKI_{SCr/UO}$ within 7-d after enrollment, had higher urinary biomarker concentrations at enrollment than all patients who did not develop $AKI_{SCr/UO}$ within 12-h after enrollment (P=0.002 for UCHI3L1 and P=0.001 for UNGAL; Additional File 4: Supplemental Figure S3). Similarly, 7-d no-AKI_{SCr} patients who were referred from the floor at ICU admission showed higher enrollment concentrations of both urinary biomarkers than all 24-h no-AKI_{SCr} patients (P=0.001 for both biomarkers; Additional File 5: Supplemental Figure S4).

Additional diagnostic performances

The AUC-ROCs for predicting $AKI_{SCr/UO}$ stage ≥ 1 within 12-h and 24-h in patients with no $AKI_{SCr/UO}$ at enrollment were markedly decreased for both UCHI3L1 and UNGAL (Table S4C). Likewise, the AUC-ROCs for predicting AKI_{SCr} stage ≥ 1 within 12-h and 24-h in patients with no AKI_{SCr} at enrollment were markedly decreased as well (Table S4C). This can be explained by less renal stress or damage in patients with AKI stage 1.

Stability of CHI3L1 in serum and urine

Storage at 4°C up to 48-h before centrifugation had no effect on the CHI3L1 concentration in both serum and urine ($P \geq 0.05$), with a mean CV ranging from 3.8 to 3.9% for serum and from 5.0 to 8.5% for urine (Table S4D).

Refreezing at -80°C followed by a second thawing step had no effect on the CHI3L1 concentration in urine ($P \geq 0.05$), even when the time between initial freezing and second thawing was 30-mo, with a mean CV ranging from 0.7 to 18.8% (Table S4E).

Partial in-house validation of the CHI3L1 ELISA

For the human CHI3L1 standards 0.25, 0.50 and 1.00 ng/ml of the ELISA, the intra-assay between-lot CV was 2.9% for the 0.25 ng/ml standard, 4.4% for the 0.50 ng/ml standard, and 5.0% for the 1.00 ng/ml standard (Table S4F). Serum samples with a low or a high CHI3L1 concentration showed an excellent intra-assay between-lot CV of 2.6% for the low and 3.2% for the high concentrations (Table S4G). For urine samples with a low CHI3L1 concentration an intra-assay between-lot CV of 4.1% was calculated (Table S4H). A mean intra-assay within-lot CV of 6.7% was obtained for serum samples with an intermediate CHI3L1 concentration. Likewise, a mean intra-assay within-lot CV of 1.9% was obtained for urine samples with a high CHI3L1 concentration.

The LOD was determined as 0.02 ng/ml, the LOQ as 0.06 ng/ml.

For all 3 serial dilution experiments, each being performed with another urine sample, the 95% CI of the slope of the linear regression equation included 1, indicating 100% recovery (Table S4I). The corresponding R^2 was systematically ≥0.99.

CONCLUSIONS REGARDING THE VALIDATION OF THE ANALYTICAL STABILITY OF CHI3L1 AND THE PARTIAL IN-HOUSE VALIDATION OF THE CHI3L1 ELISA

As an important first step, we showed that serum and urine may be stored for at least 48-h at 4°C before centrifugation without affecting the CHI3L1 stability, in analogy with the reported data for urinary neutrophil gelatinase-associated lipocalin [16]. Based on these results the study personnel could flexibly plan the handling of the samples, especially in weekends (Table S3A). When subsequently stored at -80°C, UCHI3L1 may even be measured after a second thawing step within at least 30-mo after the first freezing, in analogy with the reported data for SCHI3L1 [3, 12]. This information was required for our laboratory agenda, and is also relevant for biobanking purposes. Additionally, we can guarantee that our reported CHI3L1 concentrations are accurate and reproducible.

LIST OF ABBREVIATIONS

4PL: 4-parameter logistic; AKI: acute kidney injury; AKI_{SCr}: AKI that was diagnosed and classified by the KDIGO SCr criteria; $AKI_{SCr/UO}$: AKI that was diagnosed and classified by the KDIGO SCr and UO criteria; AUC-ROC: area under the receiver-operating characteristics curve; CHI3L1: chitinase 3-like protein 1; CI: confidence interval; CKD-EPI: Chronic Kidney Disease Epidemiology Collaboration; Conc: concentration; Cr: creatinine; CV: coefficient of variation; eGFR: estimated glomerular filtration rate; ELISA: enzyme-linked immunosorbent assay; h: hour; ICU: intensive care unit; IQR: interquartile range; KDIGO: Kidney Disease: Improving Global Outcomes; LOD: limit of detection; LOQ: limit of quantification; min: minute; mo: month; NGAL: neutrophil gelatinase-associated lipocalin; OD: optical density; PETIA: particle-enhanced turbidimetric immunoassay; R^2: coefficient of determination; SCHI3L1: serum CHI3L1; SCr: serum Cr; SD: standard deviation; SOFA: Sepsis-related Organ Failure Assessment; UCHI3L1: urinary CHI3L1; UNGAL: urinary NGAL; UO: urine output; y: year.

SUPPLEMENTAL REFERENCES

1. Remer T, Montenegro-Bethancourt G, Shi LJ: **Long-term urine biobanking: Storage stability of clinical chemical parameters under moderate freezing conditions without use of preservatives**. *Clin Biochem* 2014, **47**:307-311.
2. Gislefoss RE, Grimsrud TK, Morkrid L: **Long-term stability of serum components in the Janus Serum Bank**. *Scand J Clin Lab Invest* 2008, **68**:402-409.
3. Johansen JS, Jensen BV, Roslind A, Nielsen D, Price PA: **Serum YKL-40, a new prognostic biomarker in cancer patients?** *Cancer Epidemiol Biomarkers Prev* 2006, **15**:194-202.
4. Lippi G, Aloe R, Storelli A, Cervellin G, Trenti T: **Evaluation of NGAL Test (TM), a fully-automated neutrophil gelatinase-associated lipocalin (NGAL) immunoassay on Beckman Coulter AU 5822**. *Clin Chem Lab Med* 2012, **50**:1581-1584.
5. Parikh CR, Coca SG, Thiessen-Philbrook H, Shlipak MG, Koyner JL, Wang Z, Edelstein CL, Devarajan P, Patel UD, Zappitelli M *et al*: **Postoperative Biomarkers Predict Acute Kidney Injury and Poor Outcomes after Adult Cardiac Surgery**. *J Am Soc Nephrol* 2011, **22**:1748-1757.
6. Delong ER, Delong DM, Clarkepearson DI: **Comparing the Areas under 2 or More Correlated Receiver Operating Characteristic Curves - a Nonparametric Approach**. *Biometrics* 1988, **44**:837-845.
7. Youden WJ: **Index for rating diagnostic tests**. *Cancer* 1950, **3**:32-35.
8. Hyndman RJ, Fan YN: **Sample quantiles in statistical packages**. *Am Stat* 1996, **50**:361-365.
9. Wilson EB: **Probable Inference, the Law of Succession, and Statistical Inference**. *J Am Stat Assoc* 1927, **22**:209-212.
10. Newcombe RG: **Two-sided confidence intervals for the single proportion: Comparison of seven methods**. *Stat Med* 1998, **17**:857-872.
11. Tukey JW: **Exploratory data analysis**. Reading (MA): Addison-Wesley; 1977.
12. Hogdall EVS, Johansen JS, Kjaer SK, Price PA, Blaakjaer J, Hogdall CK: **Stability of YKL-40 concentration in blood samples**. *Scand J Clin Lab Invest* 2000, **60**:247-251.
13. Büttner J, Borth R, Boutwell JH, Broughton PMG, Bowyer RC: **Approved Recommendation (1978) on Quality-Control in Clinical-Chemistry. Part 2. Assessment of Analytical Methods for Routine Use**. *Clin Chim Acta* 1979, **98**:F145-F162.

14. Raila J, Forterre S, Kohn B, Brunnberg L, Schweigert FJ: **Effects of chronic renal disease on the transport of vitamin A in plasma and urine of dogs**. *Am J Vet Res* 2003, **64**:874-879.

15. Vincent JL, Moreno R, Takala J, Willatts S, De Mendonca A, Bruining H, Reinhart CK, Suter PM, Thijs LG: **The SOFA (Sepsis-related Organ Failure Assessment) score to describe organ dysfunction/failure. On behalf of the Working Group on Sepsis-Related Problems of the European Society of Intensive Care Medicine**. *Intensive Care Med* 1996, **22**:707-710.

16. Pedersen KR, Ravn HB, Hjortdal VE, Norregaard R, Povlsen JV: **Neutrophil Gelatinase-Associated Lipocalin (NGAL): Validation of commercially available ELISA**. *Scand J Clin Lab Invest* 2010, **70**:374-382.

SUPPLEMENTAL TABLES

Table S1 Kidney Disease: Improving Global Outcomes definition and classification of acute kidney injury.[a]

**KDIGO AKI definition**	SCr increase to ≥ 1.5 times baseline, which is known or presumed to have occurred within the prior 7-d<br>SCr increase by ≥ 0.3 mg/dl within 48-h<br>UO < 0.5 ml/kg/h for ≥ 6 consecutive h	
⇩		
**KDIGO AKI stage[b]**	SCr	UO
**1**	1a.<br>Increase to ≥ 1.5 times baseline<br>1b.<br>Increase by ≥ 0.3 mg/dl	1c.<br>< 0.5 ml/kg/h for ≥ 6 consecutive h
**2**	2a.<br>Increase to ≥ 2 times baseline	2b.<br>< 0.5 ml/kg/h for ≥ 12 consecutive h
**3**	3a.<br>Increase to ≥ 3 times baseline<br>3b.<br>Increase to ≥ 4 mg/dl<br>3c.<br>Initiation of RRT	3d.<br>< 0.3 ml/kg/h for ≥ 24 consecutive h<br>3e.<br>Anuria for ≥ 12 consecutive h

AKI: acute kidney injury; KDIGO: Kidney Disease: Improving Global Outcomes; RRT: renal replacement therapy; SCr: serum creatinine; UO: urine output.

[a]Kidney Disease: Improving Global Outcomes (KDIGO) Acute Kidney Injury Work Group: **KDIGO Clinical Practice Guideline for Acute Kidney Injury**. *Kidney Int Suppl* 2012, **2**:1-138.

[b]For staging purposes, patients should be staged according to the criterion or criteria that give(s) them the highest stage.

Table S2 STrengthening the Reporting of OBservational studies in Epidemiology statement - checklist of items that should be included in the reports of cohort studies.[a]

	Item No.	STROBE recommendation	Fulfilled?
**Title and abstract**	1	(a) Indicate the study's design with a commonly used term in the title or the abstract	☑
		(b) Provide in the abstract an informative and balanced summary of what was done and what was found	☑
**Introduction**			
Background/rationale	2	Explain the scientific background and rationale for the investigation being reported	☑
Objectives	3	State specific objectives, including any pre-specified hypotheses	☑
**Methods**			
Study design	4	Present key elements of study design early in the paper	☑
Setting	5	Describe the setting, locations, and relevant dates, including periods of recruitment, exposure, follow-up, and data collection	☑
Participants	6	(a) Give the eligibility criteria, and the sources and methods of selection of participants. Describe methods of follow-up	☑
		(b) For matched studies, give matching criteria and number of exposed and unexposed	NA
Variables	7	Clearly define all outcomes, exposures, predictors, potential confounders, and effect modifiers. Give diagnostic criteria, if applicable	☑
Data sources/ measurement	8[b]	For each variable of interest, give sources of data and details of methods of assessment (measurement). Describe comparability of assessment methods if there is more than one group	☑
Bias	9	Describe any efforts to address potential sources of bias	☑
Study size	10	Explain how the study size was arrived at	☑
Quantitative variables	11	Explain how quantitative variables were handled in the analyses. If applicable, describe which groupings were chosen and why	☑
Statistical methods	12	(a) Describe all statistical methods, including those used to control for confounding	☑
		(b) Describe any methods used to examine subgroups and interactions	☑
		(c) Explain how missing data were addressed	NA
		(d) If applicable, explain how loss to follow-up was addressed	NA

	Item No.	STROBE recommendation	Fulfilled?
		(e) Describe any sensitivity analyses	NA
**Results**			
Participants	13[b]	(a) Report numbers of individuals at each stage of study - e.g., numbers potentially eligible, examined for eligibility, confirmed eligible, included in the study, completing follow-up, and analyzed	☑
		(b) Give reasons for non-participation at each stage	☑
		(c) Consider use of a flow diagram	☑
Descriptive data	14[b]	(a) Give characteristics of study participants (e.g., demographic, clinical, social) and information on exposures and potential confounders	☑
		(b) Indicate number of participants with missing data for each variable of interest	NA
		(c) Summarize follow-up time (e.g., average and total amount)	☑
Outcome data	15[b]	Report numbers of outcome events or summary measures over time	☑
Main results	16	(a) Give unadjusted estimates and, if applicable, confounder-adjusted estimates and their precision (e.g., 95% confidence interval). Make clear which confounders were adjusted for and why they were included	☑
		(b) Report category boundaries when continuous variables were categorized	NA
		(c) If relevant, consider translating estimates of relative risk into absolute risk for a meaningful time period	NA
Other analyses	17	Report other analyses done - e.g., analyses of subgroups and interactions, and sensitivity analyses	☑
**Discussion**			
Key results	18	Summarize key results with reference to study objectives	☑
Limitations	19	Discuss limitations of the study, taking into account sources of potential bias or imprecision. Discuss both direction and magnitude of any potential bias	☑
Interpretation	20	Give a cautious overall interpretation of results considering objectives, limitations, multiplicity of analyses, results from similar studies, and other relevant evidence	☑
Generalizability	21	Discuss the generalizability (external validity) of the study results	☑
**Other information**			
Funding	22	Give the source of funding and the role of the funders for the present study and, if applicable, for the original study on which the present article	☑

	Item No.	STROBE recommendation	Fulfilled?
		is based	

No.: number; STROBE: STrengthening the Reporting of OBservational studies in Epidemiology.

[a]Vandenbroucke JP, von Elm E, Altman DG, Gotzsche PC, Mulrow CD, Pocock SJ, Poole C, Schlesselman JJ, Egger M, Initiative S: **Strengthening the reporting of observational studies in epidemiology (STROBE): Explanation and elaboration**. *PLoS Med* 2007, **4**:1628-1654.

[b]Give information separately for exposed and unexposed groups.

Table S3A Two representative work schemes for sample centrifugation in weekends.

Centrifugation on			
**Work scheme 1**			
**Sat, 10 am**		**Mon, 8 am**	
**Sample from**	**Time stored at 4°C (h) before centrifugation**	**Sample from**	**Time stored at 4°C (h) before centrifugation**
Fri, 6 pm	16	Sat, 6 pm	38
Sat, 6 am	4	Sun, 6 am	26
		Sun, 6 pm	14
		Mon, 6 am	2
**Work scheme 2**			
**Sun, 10 am**		**Mon, 8 am**	
**Sample from**	**Time stored at 4°C (h) before centrifugation**	**Sample from**	**Time stored at 4°C (h) before centrifugation**
Fri, 6 pm	40	Sun, 6 pm	14
Sat, 6 am	28	Mon, 6 am	2
Sat, 6 pm	16		
Sun, 6 am	4		

Based on our stability results (Table S4D), the responsible study coordinator had to come once a weekend: on Sat or Sun.

Table S3B Dilution of serum and urine samples for the initial measurement of chitinase 3-like protein 1 by enzyme-linked immunosorbent assay.

**Serum CRP (mg/l)**	**Estimated dilution for serum sample**	**SCr (mg/dl)**	**Estimated dilution for urine sample**
**< 10**	1/200	Increase to ≥ 1.5 times baseline, which is known or presumed to have occurred within the prior 7-d	1/5
**≥ 10**	1/500	Increase by ≥ 0.3 mg/dl within 48-h	1/10

CHI3L1: chitinase 3-like protein 1; CRP: C-reactive protein; ELISA: enzyme-linked immunosorbent assay; SCr: serum creatinine.

The dilution of a serum sample for the initial measurement of CHI3L1 by ELISA was chosen based on a patient's serum CRP, while the dilution of a urine sample for the initial measurement was chosen based on a patient's SCr.

Table S3C Evaluation of the assured dynamic range of the chitinase 3-like protein 1 enzyme-linked immunosorbent assay standard curve.

Expected standard conc. (pg/ml)	Back-calculated standard conc. (pg/ml)	Recovery (%)	Acceptable recovery (%)
**4000**	X	(X/4000)*100	70-130
**2000**	X	(X/2000)*100	70-130
**1000**	X	(X/1000)*100	70-130
**500**	X	(X/500)*100	70-130
**250**	X	(X/250)*100	70-130
**125**	X	(X/125)*100	70-130
**62.5**	X	(X/62.5)*100	70-130

conc.: concentration.

If a standard point has an unacceptable recovery while the adjacent standard points have acceptable recoveries, this point is removed from the standard curve. The effect on the novel standard curve is then reviewed.

Table S3D Re-analysis of samples with a concentration (not adjusted for dilution) outside the validated dynamic range of the chitinase 3-like protein 1 enzyme-linked immunosorbent assay standard curve.

Situation 1: narrowed validated dynamic range

$OD_{sample} > OD_{validated\ highest}$	$OD_{sample} > OD_{4000}$	Re-analyze and dilute more
	$OD_{sample} < OD_{4000}$	Re-analyze and dilute equally
$OD_{sample} < OD_{validated\ lowest}$	$OD_{sample} > OD_{62.5}$	Re-analyze and dilute equally
	$OD_{sample} < OD_{62.5}$	Re-analyze and dilute less

Situation 2: validated dynamic range of [62.5-4000]

$OD_{sample} > OD_{4000}$	Option 1	Re-analyze and dilute more
	Option 2	Do not re-analyze and report as [back-calculated $Conc_{4000}$] multiplied with [dilution factor of sample][a]<br>Implication: most likely underestimation
$OD_{sample} < OD_{62.5}$	Option 1	If less dilution is possible: re-analyze and dilute less
	Option 2	If less dilution is possible: do not re-analyze and report as missing value[a]
	Option 3	If less dilution is not possible (sample already optimal diluted): see Step 3

CHI3L1: chitinase 3-like protein 1; Conc: concentration; OD: optical density.

[a]All samples collected at enrollment were re-analyzed if needed, as CHI3L1 at enrollment is the primary dependent variable of the study.

Table S3E Adjustment of chitinase 3-like protein 1 concentrations of optimally diluted samples with optical density$_{sample}$ < optical density$_{62.5}$.

$Conc_{sample}$<br>< $LOD_{23.9}$	$Conc_{sample}$<br>< back-calculated $Conc_{62.5}$	0-23.8<br>< 23.9<br>< 43.8-81.3	$Conc_{sample}$<br>< $LOD_{23.9}$<br>< back-calculated $Conc_{62.5}$	Median between 0 and 23.9
$Conc_{sample}$<br>= $LOD_{23.9}$	$Conc_{sample}$<br>< back-calculated $Conc_{62.5}$	23.9<br>= 23.9<br>< 43.8-81.3	$Conc_{sample}$<br>= $LOD_{23.9}$<br>< back-calculated $Conc_{62.5}$	23.9
$LOD_{23.9}$<br>< $Conc_{sample}$<br>< $LOQ_{64.6}$		Option 1		
	$Conc_{sample}$<br>< back-calculated $Conc_{62.5}$	23.9<br>< 24-64.4<br>< 43.8-64.5<br>< 64.6	$LOD_{23.9}$<br>< $Conc_{sample}$<br>< back-calculated $Conc_{62.5}$<br>< $LOQ_{64.6}$	Median between 23.9 and 64.6
		Option 2		
	$Conc_{sample}$<br>≥ back-calculated $Conc_{62.5}$	23.9<br>< 43.8-64.5<br>≤ 43.8-64.5<br>< 64.6	$LOD_{23.9}$<br>< back-calculated $Conc_{62.5}$<br>≤ $Conc_{sample}$<br>< $LOQ_{64.6}$	Median between 23.9 and 64.6<br>(even if $Conc_{sample}$ falls within standard curve range)
$Conc_{sample}$<br>≥ $LOQ_{64.6}$		Option 1		
	$Conc_{sample}$<br>< back-calculated $Conc_{62.5}$	64.6<br>≤ 64.6-81.2<br>< 64.7-81.3	$LOQ_{64.6}$<br>≤ $Conc_{sample}$<br>< back-calculated $Conc_{62.5}$	Median between 23.9 and 64.6<br>(even if $Conc_{sample}$ equals or exceeds $LOQ_{64.6}$)
		Option 2		

	$Conc_{sample}$<br>≥ back-calculated $Conc_{62.5}$	64.6<br>≤ 64.6-81.3<br>≤ 64.6-X[a]	$LOQ_{64.6}$<br>≤ back-calculated $Conc_{62.5}$<br>≤ $Conc_{sample}$	Measured Conc

CHI3L1: chitinase 3-like protein 1; Conc: concentration; LOD: limit of detection; LOQ: limit of quantification; OD: optical density.

Note that the CHI3L1 concentrations are not yet corrected for dilution.

[a]X represents a concentration > 64.6 pg/ml.

Table S3F Adjustment of urinary neutrophil gelatinase-associated lipocalin concentrations before input in statistical programs.

Measured UNGAL	Reported UNGAL (ng/ml)
$< LOD_{12}$	0.1
$= LOD_{12}$	12.0
$> LOD_{12}$ and $< LOQ_{25}$	Median (LOD_{12}, LOQ_{25}) = 18.5
$\geq LOQ_{25}$	Measured UNGAL

LOD: limit of detection; LOQ: limit of quantification; UNGAL: urinary neutrophil gelatinase-associated lipocalin.

Table S4A Definition of suspected bacterial infection, of arterial hypotension, of organ dysfunction, and of shock.

Medication	Antibiotic drug	‘Yes’ or ‘No’	In paper: referred to as infection
**Suspected bacterial infection**		**If ‘Yes’**	
Medication	Antibiotic drug	‘Yes’	In paper: referred to as infection ++
Arterial hypotension	(a) Vasopressor support for at least 1-h	‘Yes’ or ‘No’	
	(b) MAP < 70 mm Hg	‘Yes’ or ‘No’	
Organ dysfunction	(a) SCr > 2.0 mg/dl	‘Yes’ or ‘No’	
	(b) Serum bilirubin (total) > 2.0 mg/dl	‘Yes’ or ‘No’	
	(c) Platelet count < 100 000/µl	‘Yes’ or ‘No’	
**Suspected bacterial infection leading to arterial hypotension or organ dysfunction**		**If at least 1x ‘Yes’**	
Medication	Antibiotic drug	‘Yes’	
Shock defined as non-responsive arterial hypotension	(a) Vasopressor support for at least 1-h	‘Yes’ or ‘No’	
	(b) MAP < 65 mm Hg and vascular filling	‘Yes’ or ‘No’	
**Suspected bacterial infection leading to shock**		**If at least 1x ‘Yes’**	

MAP: mean arterial pressure; SCr: serum creatinine.

Table S4B Experimental setup for evaluation of between-run precision or inter-assay variability by the manufacturer.

**Technician 1**		**Technician 2**		**Technician 3**		**Technician 4**	
**Reagent set 1**	**Reagent set 2**	**Reagent set 1**	**Reagent set 2**	**Reagent set 1**	**Reagent set 2**	**Reagent set 1**	**Reagent set 2**
Day 1	Day 6	Day 1	Day 6	Day 1	Day 6	Day 1	Day 6
Day 2	Day 7	Day 2	Day 7	Day 2	Day 7	Day 2	Day 7
Day 3	Day 8	Day 3	Day 8	Day 3	Day 8	Day 3	Day 8
Day 4	Day 9	Day 4	Day 9	Day 4	Day 9	Day 4	Day 9
Day 5	Day 10	Day 5	Day 10	Day 5	Day 10	Day 5	Day 10
Within-lot CV by tech 1	Within-lot CV by tech 1	Within-lot CV by tech 2	Within-lot CV by tech 2	Within-lot CV by tech 3	Within-lot CV by tech 3	Within-lot CV by tech 4	Within-lot CV by tech 4
Between-lot CV by tech 1		Between-lot CV by tech 2		Between-lot CV by tech 3		Between-lot CV by tech 4	
**Reported by manufacturer: mean of between-lot CVs**							

CV: coefficient of variation.

Day X of technician 1 is not necessarily the same as day X of technicians 2-4.

Table S4C Areas under the receiver-operating characteristics curves with 95% confidence interval of urinary chitinase 3-like protein 1 and urinary neutrophil gelatinase-associated lipocalin at enrollment for prediction of the additional endpoints.

Biomarker measurement	Time window	$AKI_{SCr/UO}$ stage ≥ 1[a]			AKI_{SCr} stage ≥ 1[b]		
		AUC-ROC	95% CI	Number of positives (%)	AUC-ROC	95% CI	Number of positives (%)
**Enrollment UCHI3L1**	**12-h**	0.614	0.534-0.691	18 (11.4)	0.647	0.568-0.721	7 (4.4)
	**24-h**	0.554	0.473-0.633	36 (22.8)	0.603	0.523-0.680	15 (9.4)
**Enrollment UNGAL**	**12-h**	0.553	0.472-0.632	18 (11.4)	0.503	0.423-0.583	7 (4.4)
	**24-h**	0.542	0.461-0.621	36 (22.8)	0.504	0.424-0.584	15 (9.4)

AKI: acute kidney injury; AUC-ROC: area under the receiver-operating characteristics curve; CI: confidence interval; KDIGO: Kidney Disease: Improving Global Outcomes; SCr: serum creatinine; UCHI3L1: urinary chitinase 3-like protein 1; UNGAL: urinary neutrophil gelatinase-associated lipocalin; UO: urine output.

[a]Based on the KDIGO SCr or UO criteria for AKI.

[b]Based on the KDIGO SCr criteria for AKI.

Table S4D Short-term stability of chitinase 3-like protein 1 in serum and urine before centrifugation.

Time between 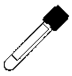 and 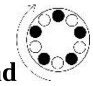 (h)	X-h[a] conc. (ng/ml)	0-h[a] conc. (ng/ml)	Mean	SD	CV (%)	Mean CV (%)	P value[b]
**Serum**							
**24**	1035.3	1060.2	1047.72	17.62	1.7	3.8	0.180
	1396.5	1514.9	1455.70	83.75	5.8		
**48**	978.3	1060.2	1019.23	57.91	5.7	3.9	0.655
	1559.7	1514.9	1537.32	31.68	2.1		
**Urine**							
**6**	1050.3	1035.8	1043.03	10.30	1.0	6.3	0.655
	577.4	679.2	628.29	71.99	11.5		
**24**	3.6	3.6	3.59	0.06	1.6	8.5	0.593
	12.5	15.7	14.13	2.27	16.0		
	1096.1	1035.8	1065.93	42.68	4.0		
	571.1	679.2	625.17	76.40	12.2		
**48**	3.9	3.6	3.77	0.20	5.2	5.0	0.715
	14.1	15.7	14.89	1.19	8.0		
	1039.2	1035.8	1037.46	2.42	0.2		
	619.0	679.2	649.08	42.59	6.6		

conc.: concentration; CV: coefficient of variation; SD: standard deviation.

[a]The concentration of a sample that was stored for X-h at 4°C before centrifugation is represented by X-h, while that of a sample that was centrifuged immediately after collection is represented by 0-h.

[b]The P values are the significance levels between samples that were immediately centrifuged and those stored for 6-, 24-, or 48-h at 4°C before centrifugation.

Table S4E Combined long-term and freeze-thaw stability of chitinase 3-like protein 1 in urine.

Time between 1st and 2nd (mo)	Conc. 2[a] (ng/ml)	Conc. 1[a] (ng/ml)	Mean	SD	CV (% minus 6.0%[b])	Mean CV (%)	P value[c]
**6**	2.3	3.0	2.65	0.48	12.3	18.8	0.655
	0.7	0.4	0.54	0.17	25.3		
**12**	4.2	3.8	4.03	0.30	1.3	0.7	0.180
	8.6	8.1	8.35	0.37	0.0[d]		
**18**	4.3	3.7	3.97	0.43	4.8	3.6	0.655
	9.5	10.7	10.12	0.85	2.4		
**24**	2.0	1.9	1.95	0.10	0.0[d]	11.9	0.655
	0.6	0.9	0.72	0.21	23.7		
**30**	14.0	11.9	12.94	1.46	5.3	2.6	0.180
	6.3	5.9	6.09	0.32	0.0[d]		

conc.: concentration; CV: coefficient of variation; SD: standard deviation.

[a]The concentration of a sample measured after 1st thawing of an aliquot is represented by 1, while that of a sample measured after 2nd thawing of an aliquot is represented by 2. The samples in this stability study were initially analyzed (conc. 1) after a period ranging from 1-9 mo.

[b]Mean inter-assay CV reported by the manufacturer = 6.0% (Table S4B).

[c]The P values are the significance levels between samples that were thawed for the 1st time and those thawed for the 2nd time.

[d]CV of (conc. 2, conc. 1) ≤ mean inter-assay CV of 6.0%.

Table S4F Within-run precision or intra-assay variability of the chitinase 3-like protein 1 enzyme-linked immunosorbent assay for standard points.

Analyte concentration (ng/ml)	Standards (n)	Repetitions (n)	Mean	SD	CV (%)	Between-lot CV with range of mean within-lot CV (%)
Standard 0.3	4	2	0.25[a]	0.00	0.9	2.9 (2.6-3.9)
			0.25[a]	0.01	3.2	
			0.23[a]	0.01	3.7	
			0.25[b]	0.01	3.9	
Standard 0.5	11	2	0.49[a]	0.02	5.1	4.4 (3.5-4.6)
			0.46[a]	0.03	6.9	
			0.52[a]	0.01	2.6	
			0.53[a]	0.02	4.2	
			0.51[b]	0.02	3.5	
			0.49[c]	0.02	3.9	
			0.46[a]	0.03	6.9	
			0.49[a]	0.01	2.6	
			0.48[d]	0.02	4.2	
			0.49[d]	0.02	4.9	
			0.49[a]	0.02	4.2	
Standard 1.0	18	2	1.01[a]	0.02	2.4	5.0 (2.4-5.8)
		2	0.95[b]	0.07	7.5	
		2	1.02[b]	0.00	0.3	
		2	0.97[b]	0.02	1.9	
		2	0.97[b]	0.03	2.7	
		2	0.94[b]	0.04	4.2	
		2	0.97[c]	0.04	3.8	
		2	0.95[c]	0.03	3.1	
		2	0.94[c]	0.03	3.5	
		2	0.98[b]	0.03	2.8	

Analyte concentration (ng/ml)	Standards (n)	Repetitions (n)	Mean	SD	CV (%)	Between-lot CV with range of mean within-lot CV (%)
		2	0.96[b]	0.10	10.3	
		2	0.97[b]	0.02	2.5	
		4	1.10[c]	0.10	9.2	
		2	0.95[c]	0.07	7.4	
		2	0.94[c]	0.07	7.7	
		2	0.93[b]	0.11	12.3	
		2	1.05[b]	0.05	4.7	
		2	0.98[b]	0.04	3.6	

CV: coefficient of variation; SD: standard deviation.

Letters in superscript indicate different lots within each group. In the 3 different groups, letter X does not necessarily represent the same lot.

Table S4G Within-run precision or intra-assay variability of the chitinase 3-like protein 1 enzyme-linked immunosorbent assay for serum samples.

Analyte concentration (ng/ml)	Serum samples (n)	Repetitions (n)	Mean	SD	CV (%)	Between-lot CV with range of mean within-lot CV (%)
**Low**	5	2	0.45[a]	0.02	3.9	2.6 (2.1-2.8)
			0.86[a]	0.01	1.2	
			0.73[a]	0.01	1.2	
			0.69[b]	0.01	2.1	
			0.95[a]	0.05	4.8	
**Intermediate > 1.0**	2	2	1.21[a]	0.02	1.8	(6.7)
		4	2.22[a]	0.26	11.6	
**High > 2.5**	3	2	3.01[a]	0.05	1.7	3.2 (1.7-3.9)
			3.08[b]	0.1	3.1	
			<u>3.24[b]</u>	<u>0.15</u>	<u>4.6</u>	

CV: coefficient of variation; OD: optical density; SD: standard deviation.

<u>Underlined results</u> represent the mean, SD and CV of the OD value (out of dynamic range of the curve).

Letters in superscript indicate different lots within each group. In the 3 different groups, letter X does not necessarily represent the same lot.

Table S4H Within-run precision or intra-assay variability of the chitinase 3-like protein 1 enzyme-linked immunosorbent assay for urine samples.

Analyte concentration (ng/ml)	Urine samples (n)	Repetitions (n)	Mean	SD	CV (%)	Between-lot CV with range of mean within-lot CV (%)
**Low**	9	2	0.21[a]	0.00	0.3	4.1 (1.6-9.3)
			0.07[a]	0.00	2.8	
			0.17[a]	0.00	1.7	
			0.18[a]	0.00	0.8	
			0.20[a]	0.01	3.7	
			0.20[b]	0.03	14.8	
			0.09[c]	0.00	1.6	
			0.94[a]	0.07	7.0	
			0.70[b]	0.03	3.9	
**High > 2.5**	2	2	2.52[a]	0.06	2.3	(1.9)
			2.74[a]	0.04	1.4	

CV: coefficient of variation; OD: optical density; SD: standard deviation.

Underlined results represent the mean, SD and CV of the OD value (out of dynamic range of the curve).

Letters in superscript indicate different lots within each group. In the 2 different groups, letter X does not necessarily represent the same lot.

Table S4I Assessment of the enzyme-linked immunosorbent assay linearity for urinary chitinase 3-like protein 1.

Urine sample	Dilution	Expected conc.	Measured conc.	Linear regression graph	Slope (95% CI)	R^2
**1**	1/2	0.953	0.953	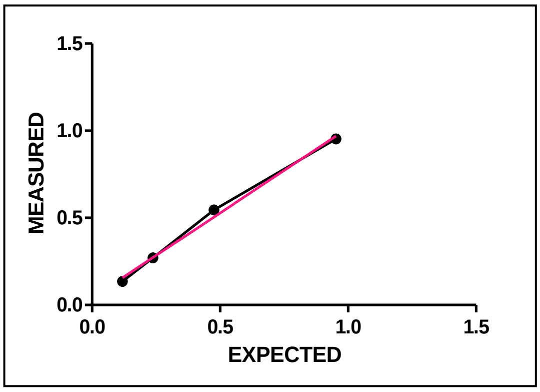	0.98 (0.75-1.21)	0.99
	1/4	0.476	0.545			
	1/8	0.238	0.271			
	1/16	0.119	0.136			
**2**	1/2	2.248	2.248	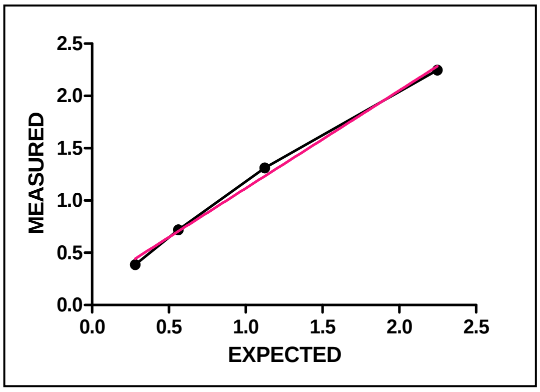	0.94 (0.72-1.15)	0.99
	1/4	1.124	1.312			
	1/8	0.562	0.720			
	1/16	0.281	0.387			
**3**	1/2	2.569	2.569	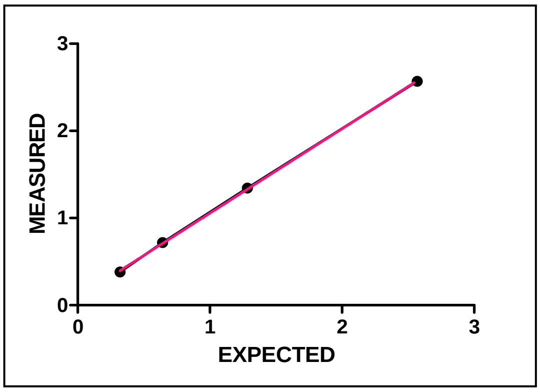	0.97 (0.93-1.01)	1.00
	1/4	1.284	1.345			
	1/8	0.642	0.719			
	1/16	0.321	0.381			

CI: confidence interval; conc.: concentration; OD: optical density; $R^{2:}$ coefficient of determination.

The pink lines represent the best fitted linear regression lines.

Table S5: Sharpened clinical phenotype analysis with areas under the receiver-operating characteristics curves of urinary chitinase 3-like protein 1 and urinary neutrophil gelatinase-associated lipocalin at enrollment.

Biomarker measurement	Time window	$AKI_{SCr/UO}$ stage ≥ 2[a]			AKI_{SCr} stage ≥ 2[b]		
		AUC-ROC	95% CI	Number of positives/total N	AUC-ROC	95% CI	Number of positives/total N
Enrollment UCHI3L1	12-h	0.882	0.817-0.930	2/142	0.879	0.817-0.926	2/155
	24-h	0.631	0.541-0.715	4/126	0.879	0.815-0.927	2/147
Enrollment UNGAL	12-h	0.850	0.780-0.904	2/142	0.856	0.791-0.907	2/155
	24-h	0.654	0.564-0.736	4/126	0.852	0.784-0.905	2/147

AKI: acute kidney injury; AUC-ROC: area under the receiver-operating characteristics curve; CI: confidence interval; KDIGO: Kidney Disease: Improving Global Outcomes; SCr: serum creatinine; UCHI3L1: urinary chitinase 3-like protein 1; UNGAL: urinary neutrophil gelatinase-associated lipocalin; UO: urine output.

[a]Based on the KDIGO SCr or UO criteria for AKI.

[b]Based on the KDIGO SCr criteria for AKI.

Table S6: Spearman's coefficients of rank correlation for UCHI3L1 and UNGAL, both measured at enrollment, in the total analysis cohort, in subgroups separated by AKI stage at enrollment, and in subgroups separated by AKI stage within 12-h and 24-h after enrollment.

Time	Group	Spearman's coefficient of rank correlation	95% CI	Number of patients
Enrollment	All	0.615	0.515-0.698	181

Time	Subgroup	$AKI_{SCr/UO}$[a] Spearman's coefficient of rank correlation	$AKI_{SCr/UO}$[a] 95% CI	$AKI_{SCr/UO}$[a] Number of patients	AKI_{SCr}[b] Spearman's coefficient of rank correlation	AKI_{SCr}[b] 95% CI	AKI_{SCr}[b] Number of patients
Enrollment	Stage 0[c]	0.600	0.489-0.691	158	0.595	0.484-0.686	160
	Stage 1	0.532	0.153-0.774	23	0.575	0.191-0.807	21

Time window starting from enrollment	Subgroup	$AKI_{SCr/UO}$[a] Spearman's coefficient of rank correlation	$AKI_{SCr/UO}$[a] 95% CI	$AKI_{SCr/UO}$[a] Number of patients	AKI_{SCr}[b] Spearman's coefficient of rank correlation	AKI_{SCr}[b] 95% CI	AKI_{SCr}[b] Number of patients
12-h	Stage 0[c] or 1	0.601	0.497-0.688	175	0.596	0.492-0.683	177
24-h		0.590	0.483-0.680	172	0.589	0.483-0.678	176
12-h	Stage 2 or 3	0.543	-0.480-0.940	6	0.800	-0.697-0.996	4
24-h		0.800	0.290-0.956	9	0.900	0.086-0.993	5

AKI: acute kidney injury; CI: confidence interval; KDIGO: Kidney Disease: Improving Global Outcomes; SCr: serum creatinine; UCHI3L1: urinary chitinase 3-like protein 1; UNGAL: urinary neutrophil gelatinase-associated lipocalin; UO: urine output.

[a]Based on the KDIGO SCr or UO criteria for AKI.

[b]Based on the KDIGO SCr criteria for AKI.

[c]No AKI

Table S7: Youden index with the associated criterion value of the urinary biomarker.

Biomarker measurement	Time window	AKI$_{SCr/UO}$ stage ≥ 2[a]				AKI$_{SCr}$ stage ≥ 2[b]			
		J	Sens. (%)	Spec. (%)	Crit. value (ng/ml)	J	Sens. (%)	Spec. (%)	Crit. value (ng/ml)
**Enrollment UCHI3L1**	**12-h**	0.651	83.3	81.7	> 7.6	0.814	100.0	81.4	> 7.6
	**24-h**	0.486	66.7	82.0	> 7.6	0.818	100.0	81.8	> 7.6
**Enrollment UNGAL**	**12-h**	0.467	66.7	80.0	> 139.0	0.802	100.0	80.2	> 139.0
	**24-h**	0.440	66.7	77.3	> 111.0	0.807	100.0	80.7	> 139.0

AKI: acute kidney injury; Crit.: criterion; J: Youden index defined as the maximum of [sensitivity plus specificity minus 1]; KDIGO: Kidney Disease: Improving Global Outcomes; SCr: serum creatinine; UCHI3L1: urinary chitinase 3-like protein 1; UNGAL: urinary neutrophil gelatinase-associated lipocalin; Sens.: sensitivity; Spec.: specificity; UO: urine output.

[a]Based on the KDIGO SCr or UO criteria for AKI.

[b]Based on the KDIGO SCr criteria for AKI.

Table S8: Proportion of patients with a concomitant very high serum chitinase 3-like protein 1 in the groups of patients who did not develop acute kidney injury and either presented with or without an increased urinary chitinase 3-like protein 1 at enrollment.

Group	N (%)	Subgroup of a or b	N (%)	Subgroup of c, d, e, or f	N (%)
(a) No $AKI_{SCr/UO}$ within 7-d after enrollment[a]	95 (100)	(c) UCHI3L1 > 7.6 ng/ml[b]	15 (16)	(g) SCHI3L1 > 2000 ng/ml	6 (40)
		(d) UCHI3L1 normal	80 (84)	(h) SCHI3L1 > 2000 ng/ml	2 (3)
(b) No AKI_{SCr} within 7-d after enrollment[c]	120 (100)	(e) UCHI3L1 > 7.6 ng/ml[d]	18 (15)	(i) SCHI3L1 > 2000 ng/ml	6 (33)
		(f) UCHI3L1 normal	102 (85)	(j) SCHI3L1 > 2000 ng/ml	5 (5)

AKI: acute kidney injury; KDIGO: Kidney Disease: Improving Global Outcomes; SCHI3L1: serum chitinase 3-like protein 1; SCr: serum creatinine; UCHI3L1: urinary chitinase 3-like protein 1; UO: urine output.

[a]Based on the KDIGO SCr or UO criteria for AKI.

[b]Criterion value of UCHI3L1 associated with the Youden index for predicting $AKI_{SCr/UO}$ stage ≥2 within 12-h after enrollment.

[c]Based on the KDIGO SCr criteria for AKI.

[d]Criterion value of UCHI3L1 associated with the Youden index for predicting AKI_{SCr} stage ≥2 within 24-h after enrollment.

SUPPLEMENTAL FIGURE LEGENDS

Figure S1 Area under the receiver-operating characteristics curve (AUC-ROC) with 95% confidence interval (CI) of **(A)** urinary chitinase 3-like protein 1 (UCHI3L1) and **(B)** urinary neutrophil gelatinase-associated lipocalin (UNGAL) at enrollment for predicting acute kidney injury (AKI) stage ≥2 based on the Kidney Disease: Improving Global Outcomes (KDIGO) serum creatinine (SCr) or urine output (UO) criteria ($AKI_{SCr/UO}$) within 12-h in different subgroups of patients.

The dotted vertical lines delineate the AUC-ROC with 95% CI in the analysis cohort. The total number of patients in each of the 9 subgroups (top-down) was 71, 134, 73, 108, 43, 138, 35, 146, and 122. The definition of infection ++ is outlined in Table S4A. eGFR: estimated glomerular filtration rate (calculated with the Chronic Kidney Disease Epidemiology Collaboration formula); ER: emergency room; M: medical; OH: other hospital; OR: operating room; S: surgical; SOFA: Sepsis-related Organ Failure Assessment.

Note: Additional File 2 is a TIFF file of Figure S1.

Figure S2 Area under the receiver-operating characteristics curve (AUC-ROC) with 95% confidence interval (CI) of **(A)** urinary chitinase 3-like protein 1 (UCHI3L1) and **(B)** urinary neutrophil gelatinase-associated lipocalin (UNGAL) at enrollment for predicting acute kidney injury (AKI) stage ≥2 based on the Kidney Disease: Improving Global Outcomes (KDIGO) serum creatinine (SCr) criteria (AKI_{SCr}) within 24-h in different subgroups of patients.

The dotted vertical lines delineate the AUC-ROC with 95% CI in the analysis cohort. The total number of patients in each of the 8 subgroups (top-down) was 71, 110, 134, 73, 43, 35, 146, and 122. The definition of infection ++ is outlined in Table S4A. eGFR: estimated glomerular filtration rate (calculated with the Chronic Kidney Disease Epidemiology Collaboration formula); S: surgical; SOFA: Sepsis-related Organ Failure Assessment.

Note: Additional File 3 is a TIFF file of Figure S2.

Figure S3 Distribution of **(A)** urinary chitinase 3-like protein 1 (UCHI3L1) and **(B)** urinary neutrophil gelatinase-associated lipocalin (UNGAL) at enrollment in the 8 selected subgroups of patients who did not develop acute kidney injury (AKI) based on the Kidney Disease: Improving Global Outcomes serum creatinine (SCr) or urine output (UO) criteria (no-$AKI_{SCr/UO}$) within 7-d after enrollment, compared to the distribution in all 12-h no-$AKI_{SCr/UO}$ patients, and in all those maximally reaching $AKI_{SCr/UO}$ stages 1, 2, or 3 within 12-h after enrollment.
The total number of patients in each group (left-right) was 33, 16, 39, 56, 16, 79, 8, 56, 140, 35, 4, and 2. The definition of infection ++ is outlined in Table S4A. eGFR: estimated glomerular filtration rate (calculated with the Chronic Kidney Disease Epidemiology Collaboration formula); ER: emergency room; OH: other hospital; OR: operating room; SOFA: Sepsis-related Organ Failure Assessment.

Note: Additional File 4 is a TIFF file of Figure S3.

Figure S4 Distribution of **(A)** urinary chitinase 3-like protein 1 (UCHI3L1) and **(B)** urinary neutrophil gelatinase-associated lipocalin (UNGAL) at

enrollment in the 8 selected subgroups of patients who did not develop acute kidney injury (AKI) based on the Kidney Disease: Improving Global Outcomes serum creatinine (SCr) criteria (no-AKI_{SCr}) within 7-d after enrollment, compared to the distribution in all 24-h no-AKI_{SCr} patients, and in all those maximally reaching AKI_{SCr} stages 1, 2, or 3 within 24-h after enrollment.

The total number of patients in each group (left-right) was 49, 28, 45, 75, 22, 98, 15, 76, 145, 31, 2, and 3. The definition of infection ++ is outlined in Table S4A. eGFR: estimated glomerular filtration rate (calculated with the Chronic Kidney Disease Epidemiology Collaboration formula); ER: emergency room; OH: other hospital; OR: operating room; SOFA: Sepsis-related Organ Failure Assessment.

Note: Additional File 5 is a TIFF file of Figure S4.
